# Integrative proteomics and phosphoproteomics in pulmonary arterial hypertension

**DOI:** 10.1038/s41598-019-55053-6

**Published:** 2019-12-09

**Authors:** Weiling Xu, Suzy A. A. Comhair, Ruoying Chen, Bo Hu, Yuan Hou, Yadi Zhou, Lori A. Mavrakis, Allison J. Janocha, Ling Li, Dongmei Zhang, Belinda B. Willard, Kewal Asosingh, Feixiong Cheng, Serpil C. Erzurum

**Affiliations:** 1 Lerner Research Institute, Cleveland Clinic, Cleveland, Ohio United States of America; 2 Respiratory Institute, Cleveland Clinic, Cleveland, Ohio United States of America

**Keywords:** Respiratory tract diseases, Mechanisms of disease, Protein-protein interaction networks

## Abstract

Pulmonary arterial endothelial cells (PAEC) are mechanistically linked to origins of pulmonary arterial hypertension (PAH). Here, global proteomics and phosphoproteomics of PAEC from PAH (*n* = 4) and healthy lungs (*n* = 5) were performed using LC-MS/MS to confirm known pathways and identify new areas of investigation in PAH. Among PAH and control cells, 170 proteins and 240 phosphopeptides were differentially expressed; of these, 45 proteins and 18 phosphopeptides were located in the mitochondria. Pathologic pathways were identified with integrative bioinformatics and human protein-protein interactome network analyses, then confirmed with targeted proteomics in PAH PAEC and non-targeted metabolomics and targeted high-performance liquid chromatography of metabolites in plasma from PAH patients (*n* = 30) and healthy controls (*n* = 12). Dysregulated pathways in PAH include accelerated one carbon metabolism, abnormal tricarboxylic acid (TCA) cycle flux and glutamate metabolism, dysfunctional arginine and nitric oxide pathways, and increased oxidative stress. Functional studies in cells confirmed abnormalities in glucose metabolism, mitochondrial oxygen consumption, and production of reactive oxygen species in PAH. Altogether, the findings indicate that PAH is typified by changes in metabolic pathways that are primarily found in mitochondria.

## Introduction

Pulmonary arterial hypertension (PAH) is a fatal disease characterized by impaired regulation of pulmonary hemodynamics and vascular growth. Endothelial cell dysfunction in the arteries of PAH lungs is mechanistically linked to the pathobiology of PAH^1–5^. For example, the endothelial production of the critical vasodilator nitric oxide (NO) is deficient in PAH^4,6–9^ due to phosphorylation inactivation of endothelial NO synthase (NOS3)^10^. Pulmonary arterial endothelial cells (PAEC) derived from human PAH lungs continue to exhibit a pathologic phenotype, including decreased NO production. The cells also manifest an abnormal metabolic phenotype that is characterized by decreased mitochondrial respiration, significantly higher glycolytic rate, apoptosis resistance, increased cell proliferation, altered hypoxia sensing, and increased oxidative stress^2–4^. Cultured primary PAEC are a valid model system to investigate the pathophysiology of PAH even after multiple passages *ex vivo* as they accurately reflect endothelial cells in vascular lesions of PAH lungs *in vivo*^2,3,10–13^.

Until recently, traditional approaches have been used to focus on a single molecule and/or pathway to investigate PAH, similar to investigations of all complex disease phenotypes. However, recent advances in proteomic and phosphoproteomic methodologies and human interactome networks offer an unbiased approach for the identification and quantification of many proteins and pathways in disease pathology^14^. Alterations in the proteomes of plasma, lung tissues, and pulmonary arterial smooth muscle cells from PAH patients were found to be associated with disease progression, poor survival, and clinical risk^15–17^. Metabolomics, the quantification of small biochemicals in plasma and tissues, can also provide new insights into the complex biochemical processes of PAH and reveal relative activities of pathways. Metabolomic analyses revealed metabolite alterations in PAEC and pulmonary arterial smooth muscle cells relevant to dysregulated vascular metabolism and disease pathogenesis^18,19^. To expand understanding of pathologic molecular mechanisms, we hypothesized that integrative analyses of protein expression and phosphorylation levels in endothelial cells would reveal pathways important to the origins of PAH. To test this, we used LC-MS/MS approach to analyze protein expression and phosphorylation levels in PAEC from PAH (*n* = 4) and controls (*n* = 5). We identified dysregulated pathways in PAH with integrative bioinformatics and human protein-protein interactome network analyses and confirmed related molecules and pathways with nontargeted metabolomics and targeted high-performance liquid chromatography (HPLC) of metabolites in plasma from PAH (*n* = 30) and healthy controls (*n* = 12).

## Results and Discussion

### Human pulmonary arterial endothelial cells

PAEC were derived from 15 individuals with PAH undergoing lung transplantation [age 33 ± 4 years; race, 11 white, 1 Asian, and 3 unknown; gender, 5 men, 9 women, and 1 unknown] and from donor lungs not used for transplantation from 14 individuals [age 43 ± 4 years; race, 9 white and 5 unknown; gender, 4 men, 9 women, and 1 unknown]. PAH was clinically confirmed by right heart catheterization [pulmonary arterial pressure (PAP) mm Hg, 62 ± 3; pulmonary vascular resistance (PVR) Wood units, 10 ± 1] and by pathologic review of explanted lungs.

### Whole proteomics of PAH and healthy controls

**Figure 1:**
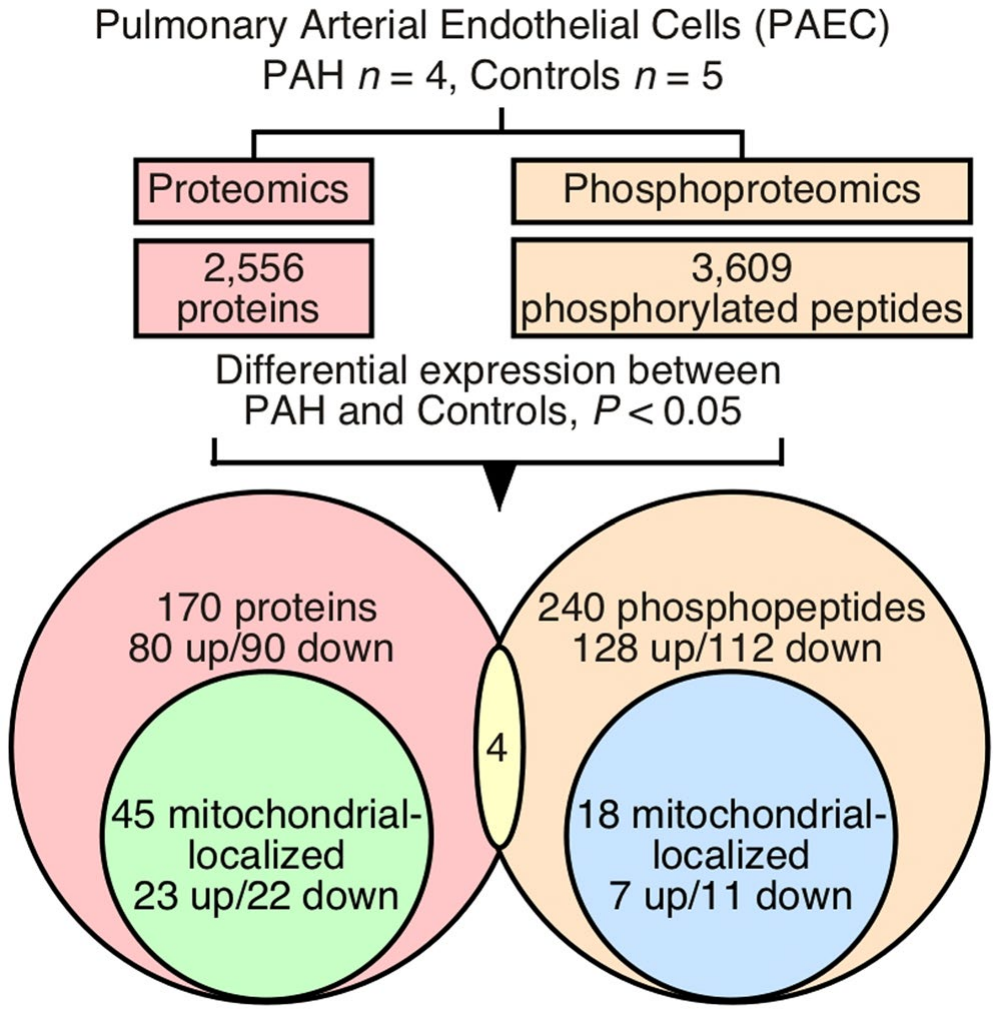
Workflow diagram summarizing proteomic and phosphoproteomic results in pulmonary arterial endothelial cells (PAEC) from 4 pulmonary arterial hypertension (PAH) patients and 5 control lungs. Only 4 differentially expressed proteins were also differentially expressed as phosphoproteins, and none of them were mitochondrial.

**Figure 2:**
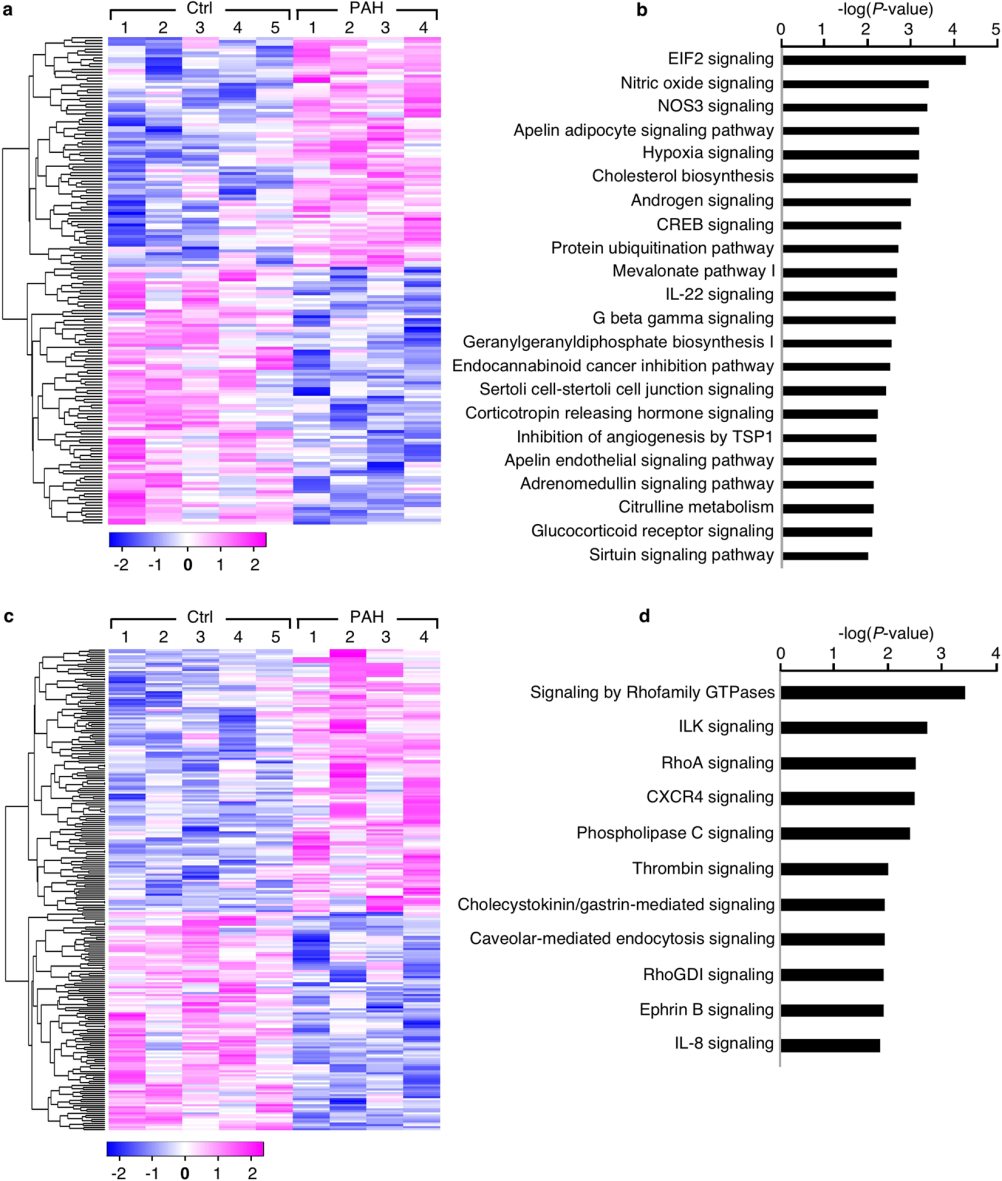
Differentially expressed proteins and phosphoproteins related to biological pathways and signaling networks in PAH PAEC. (**a**) Heatmap clustering differentially expressed proteins between PAH PAEC (PAH, *n* = 4) and healthy control PAEC (Ctrl, *n* = 5). Expression levels in PAH relative to control are represented on a continuous scale from blue (lowest) to pink (highest). (**b**) Top canonical pathways predicted by Ingenuity Pathway Analysis (IPA) in differentially expressed proteins between PAH and controls. (**c**) Heatmap clustering differentially expressed phosphopeptides between PAH PAEC (PAH, *n* = 4) and healthy control PAEC (Ctrl, *n* = 5). Expression levels in PAH relative to control are represented on a continuous scale from blue (lowest) to pink (highest). (**d**) Top canonical pathways predicted by IPA in differentially expressed phosphoproteins between PAH and control PAEC.

To identify protein expression and phosphorylation in PAH, primary PAEC derived from PAH lungs (*n* = 4) and control lungs (*n* = 5) were analyzed (Fig. 1). A total of 2,556 proteins were identified by global LC-MS/MS analysis (Supplementary Table S1). In total, 170 proteins were significantly different between PAH and control PAEC (*P* < 0.05). Among them, 80 proteins were upregulated in PAH, while 90 proteins were downregulated in PAH (Figs. 1 and 2a, Supplementary Table S2). Consistent with the high purity of PAEC cultures, proteins expressed in smooth muscle or fibroblasts [SM22α, myosin heavy chain and fibroblast-specific protein 1 (FSP1)] were undetectable in PAH or control endothelial cells. To confirm discovery findings, 22 proteins among 170 differentially expressed proteins along with actin and tubulin were picked to perform targeted analyses in 5 PAH PAEC from 5 different patients compared with control cells. Expression of actin (ACTG1) or tubulin (TUBB) was similar between PAH PAEC (*n* = 5) and controls (*n* = 5) (ACTG1, *P* = 0.8; TUBB, *P* = 0.8; two-tailed *t*-test). Twelve of 22 proteins (55%) had the same direction of differential expression between global and targeted proteomics. Within targeted analyses, 8 of these 22 proteins (36.4%) were significantly differentially expressed (*P* < 0.05, one-tailed) (Supplementary Table S2).

Differentially expressed proteins were analyzed using Ingenuity Pathway Analysis (IPA, QIAGEN) in which proteins are analyzed as a network using canonical pathways, predicted upstream regulators, biological functions and disease and functional networks. IPA analysis revealed significant differences in 37 canonical pathways, 11 upstream regulators, 22 biological functions, and 7 disease and functional networks between PAH and control PAEC (Supplementary Tables S3–S6). Canonical pathways predicted by IPA included eukaryotic initiation factor (EIF) 2 signaling, NO and NOS3 signaling, apelin, and hypoxia signaling (Fig. 2b, Supplementary Table S3), all of which have been previously identified^2,5,10,12,20–22^. IPA upstream regulator analysis showed that cell division cycle 73 (CDC73), microRNA 223 (mir-223), endothelial PAS domain protein 1 (EPAS1, HIF-2α), complement C1q binding protein (C1QBP), epidermal growth factor receptor (EGFR), Wilms tumor-1 (WT1), and others were significantly different in PAH PAEC (Supplementary Table S4). Abnormal biological functions such as cell death and survival (e.g., apoptosis, necrosis, and cell death), cellular compromise and inflammatory response, cellular movement, protein synthesis, and RNA post-transcriptional modification were found to be significantly different in PAH PAEC (Supplementary Table S5) and confirmed previous findings by traditional methods^1–5^. Changes in seven disease and functional networks (Supplementary Table S6) including hematological system development and function, and cardiovascular system development and function [network 1], cellular compromise, cellular function and maintenance, and cancer [network 2], cellular movement, cell-to-cell signaling and interaction, cell cycle, organismal injury and abnormalities, tissue morphology, cell death and survival, and cellular development were associated with PAH.

Eukaryotic initiation factor (EIF) 2 is required for protein synthesis and initiator binding of tRNA to the ribosome and plays a critical role in vascular remodeling and proliferation of pulmonary arterial vascular smooth muscle cells in hypoxia-induced pulmonary hypertension^20,23,24^. Here, EIF2 signaling-related proteins, including EIF2A, EIF3C, EIF4B, EIF4G3, EIF5B, 78 kDa glucose-regulated protein (HSPA5), and 60S ribosomal protein L3 (RPL3), were significantly upregulated, while RAC-alpha serine/threonine-protein kinase (AKT1) and mitogen-activated protein kinase 1 (MAPK1) were significantly downregulated in PAH (Supplementary Tables S2 and S3). As predicted by IPA analysis, EIF2 signaling-related proteins participate in biological functions of apoptosis, necrosis, cell death and protein synthesis (Supplementary Table S5) and are involved in the networks of cellular compromise, cellular function and maintenance, and cancer (Supplementary Table S6).

Apelin is the endogenous ligand for the G-protein-coupled apelin receptor that is expressed at the surface of the endothelium. Apelin causes NO-dependent arterial vasodilation through NOS3 activation at transcriptional and translational levels. Here, all apelin signaling-related proteins including AKT1, calmodulin-3 (CALM3), guanine nucleotide-binding protein subunit alpha-11 (GNA11), MAPK1, microsomal glutathione S-transferase 1 (MGST1), NOS3, PRKACA, and SOD1, were significantly reduced in PAH PAEC when compared to control PAEC (Supplementary Table S2). These effects might cause phosphorylation inactivation of NOS3 and less NO^10,22^. The findings confirm prior work of Chun *et al*. on this pathway in PAH^12^. Other apelin-mediated signaling pathways related to adipocytes, endothelial cells, muscles, and cardiomyocytes were also significantly downregulated in PAH PAEC as compared to control PAEC (Supplementary Table S3). Downregulated apelin signaling pathway are likely to affect β-adrenergic receptor signaling and lead to dysregulated vascular homeostasis and cardiovascular function in PAH^13,25–27^.

### Phosphoproteome of PAH and control PAEC

The characterization of the complex regulatory circuits underlying cell response to external and internal stimuli is still limited by our inability to describe the phosphorylation network on a global scale although protein phosphorylation is known to modulate a wide variety of processes. A total of 3,609 phosphopeptides derived from 1,411 phosphoproteins were identified by LC-MS/MS using phosphoserine and phosphothreonine enrichment approach (Supplementary Table S7). In total, 240 phosphopeptides derived from 202 phosphoproteins were significantly different between PAH and control PAEC (*P* < 0.05) (Supplementary Table S8). Among them, 128 phosphopeptides were upregulated, while 112 phosphopeptides were downregulated in PAH PAEC (Figs. 1 and 2c, Supplementary Table S8). Most of these have not been previously investigated in PAH.

Differentially expressed phosphoproteins were analyzed using IPA (Supplementary Tables S9–S12). Eleven canonical pathways were predicted by IPA with the top pathways being signaling by Rho family GTPases, ILK signaling, RhoA signaling, and C-X-C-motif Chemokine Receptor-4 (CXCR4) signaling (Fig. 2d, Supplementary Table S9). IPA analysis also predicted seven master regulators with estrogen receptor-beta (ESR2) as the top regulator (Supplementary Table S10) and showed that PAH PAEC had abnormal biological functions in the categories of cancer and organismal injury and abnormalities (Supplementary Table S11). Furthermore, the differentially expressed phosphoproteins in PAH PAEC were associated with nine top disease and functional networks (Supplementary Table S12), including networks of RNA post-transcriptional modification, cellular development, cellular growth and proliferation [network 1], cancer, organismal injury and abnormalities, endocrine system disorders [network 2], gene expression, cell morphology, cell death and survival, cellular assembly and organization, cellular function and maintenance, organ morphology, organismal development, hematological disease, immunological disease, hematological system development and function, lymphoid tissue structure and development, and infectious diseases.

Of the 170 differentially expressed proteins and the 240 differentially expressed phosphopeptides, only 4 proteins were both differentially expressed and had significantly different phosphorylation, i.e., eukaryotic translation initiation factor 5B (EIF5B), trans-Golgi network integral membrane protein 2 (TGOLN2), polyadenylate-binding nuclear protein 1 (PABPN1), and zinc finger CCCH domain-containing protein 4 (ZC3H4) (Fig. 1, Supplementary Tables S2 and S8).

EIF5B is responsible for catalyzing the formation of the ribosomal initiation complex for translation. Phosphorylation of EIF5B enhances ribosomal RNA processing^28^. Upregulated EIF5B protein expression but downregulated EIF5B phosphorylation may have effects on RNA translation in PAH PAEC (Proteomics, PAH/Control, fold-change [FC] = 1.3075, *P* = 0.01; Phosphoproteomics, PAH/Control, FC = 0.3214, *P* = 0.001).

TGOLN2 is a membrane protein localized to the trans-Golgi network that plays a role in exocytic vesicle formation. TGOLN2 protein levels were higher in PAH PAEC but were less phosphorylated (Proteomics, PAH/Control, FC = 1.4642, *P* = 0.04; Phosphoproteomics, PAH/Control, FC = 0.5661, *P* = 0.008). The effect of phosphorylation on TGOLN2 is unknown. The differential expression of TGOLN2 in PAH PAEC suggests that the sorting and secreting of proteins in the Golgi is altered in PAH.

PABPN1 binds to poly A tails of nascent RNA and stimulates polyadenylation, which increases message stability and decreases alternative cleavage^29^. PABPN1 protein levels were significantly lower in PAH than control PAEC but were more phosphorylated (Proteomics, PAH/Control, FC = 0.6922, *P* = 0.04; Phosphoproteomics, PAH/Control, FC = 1.5112, *P* = 0.02). Because phosphorylated PABPN1 is implicated in double-strand break repair, the findings suggest potentially greater ongoing DNA damage/repair in PAH PAEC^29^.

ZC3H4 is a zinc finger protein that is implicated in inflammation and epithelial-to-mesenchymal transition in fibrosis^30^. PAH PAEC had higher levels of ZC3H4 protein and greater protein phosphorylation than control PAEC (Proteomics, PAH/Control, FC = 1.5377, *P* = 0.0003; Phosphoproteomics, PAH/Control, FC = 1.5372, *P* = 0.03). This suggests that PAH PAEC may have greater capacity to contribute to fibrogenesis^30^.

### Mitochondrial proteomes and phosphoproteomes

Previously we and others have shown that glucose metabolism and mitochondrial respiration are altered in PAH patients and PAH PAEC^1,2,4,5,31^. Mitochondria have their own circular DNA (mtDNA), which contains 13 genes that encode proteins essential for oxidative phosphorylation. However, most mitochondrial proteins are encoded by nuclear DNA (MT-nDNA) and are imported to the mitochondria^32^. We identified 670 mitochondrial proteins (Supplementary Table S13) with 45 proteins significantly different between PAH and controls PAEC (*P* < 0.05). Among them, 23 were upregulated while 22 were downregulated in PAH PAEC when compared to control PAEC (Fig. 1, Supplementary Table S14). Phosphoproteomics identified 366 phosphopeptides derived from 154 mitochondrial proteins (Supplementary Table S15). Eighteen phosphopeptides derived from 18 mitochondrial proteins were significantly different between PAH and control PAEC (*P* < 0.05). Among them, 7 were upregulated in PAH while 11 were downregulated (Fig. 1, Supplementary Table S16). To our knowledge, nearly half of them have not been previously reported in PAH. The search tool for the retrieval of interacting genes/proteins (STRING) identified these proteins in biological processes including organonitrogen compound processes, metabolic processes of organic substance, small molecule, cellular and primary, cellular component organization, and response to stress identified in the mitochondrial proteome and phosphoproteome are listed in Supplementary Tables S17 and S18, respectively. Proteins related to organonitrogen compound biosynthetic process, which maintains a state or activity of a cell, were significantly altered in PAH, e.g., delta-1-pyrroline-5-carboxylate synthase (ALDH18A1), Golgi phosphoprotein 3 (GOLPH3), mitochondrial 28S ribosomal protein (MRPS) 28, MRPS31, MRPS7, mitochondrial monofunctional C1-tetrahydrofolate synthase (MTHFD1L), nucleoside diphosphate kinase A (NME1), RPL3, mitochondrial serine hydroxymethyltransferase (SHMT2), stomatin-like protein 2, mitochondrial (STOML2), and mitochondrial tyrosine–tRNA ligase (YARS2) were significantly upregulated, and AKT1, microsomal glutathione S-transferase 1 (MGST1), mannosyl-oligosaccharide glucosidase (MOGS), tricarboxylate transport protein, mitochondrial (SLC25A1), and v-type proton ATPase 116 kDa subunit a isoform 3 (TCIRG1) were significantly downregulated in PAH (Supplementary Table S14 and S17). Small molecule metabolic process involving low molecular weight, monomeric and non-encoded molecules was changed in PAH. Acetyl-CoA acetyltransferase (ACAT2), ALDH18A1, mitochondrial enoyl-CoA delta isomerase 1 (ECI1), MTHFD1L, NADH dehydrogenase [ubiquinone] 1 beta subcomplex subunit 7 (NDUFB7), NME1, SHMT2, STOML2 and YARS2 were significantly upregulated, while AKT1, CDP-diacylglycerol–inositol 3-phosphatidyltransferase (CDIPT) 3-beta-hydroxysteroid-Delta(8),Delta(7)-isomerase (EBP), isopentenyl-diphosphate Delta-isomerase 1 (IDI1), MAPK1 NOS3, SLC25A1, and TCIRG1 were significantly downregulated (Supplementary Table S14 and S17), suggesting metabolic pathways and processes involving small molecules were different in PAH as compared to healthy controls. Furthermore, stress-related proteins had significantly increased phosphorylation in transcription factor AP-1 (JUN) and vimentin (VIM), but decreased phosphorylation in annexin A1 (ANXA1), autophagy-related protein 16-1 (ATG16L1), inositol 1,4,5-trisphosphate receptor type 3 (ITPR3), serine/threonine-protein kinase mTOR (MTOR), myosin-10 (MYH10), poly [ADP-ribose] polymerase 4 (PARP4), NAD-dependent protein deacetylase sirtuin-1 (SIRT1), DNA topoisomerase 2-alpha (TOP2A), and tumor suppressor p53-binding protein 1 (TP53BP1) (Supplementary Table S16 and S18), indicating that cellular response to stress was significantly different in PAH endothelial cells.

### Human protein-protein interactome network analyses

**Figure 3:**
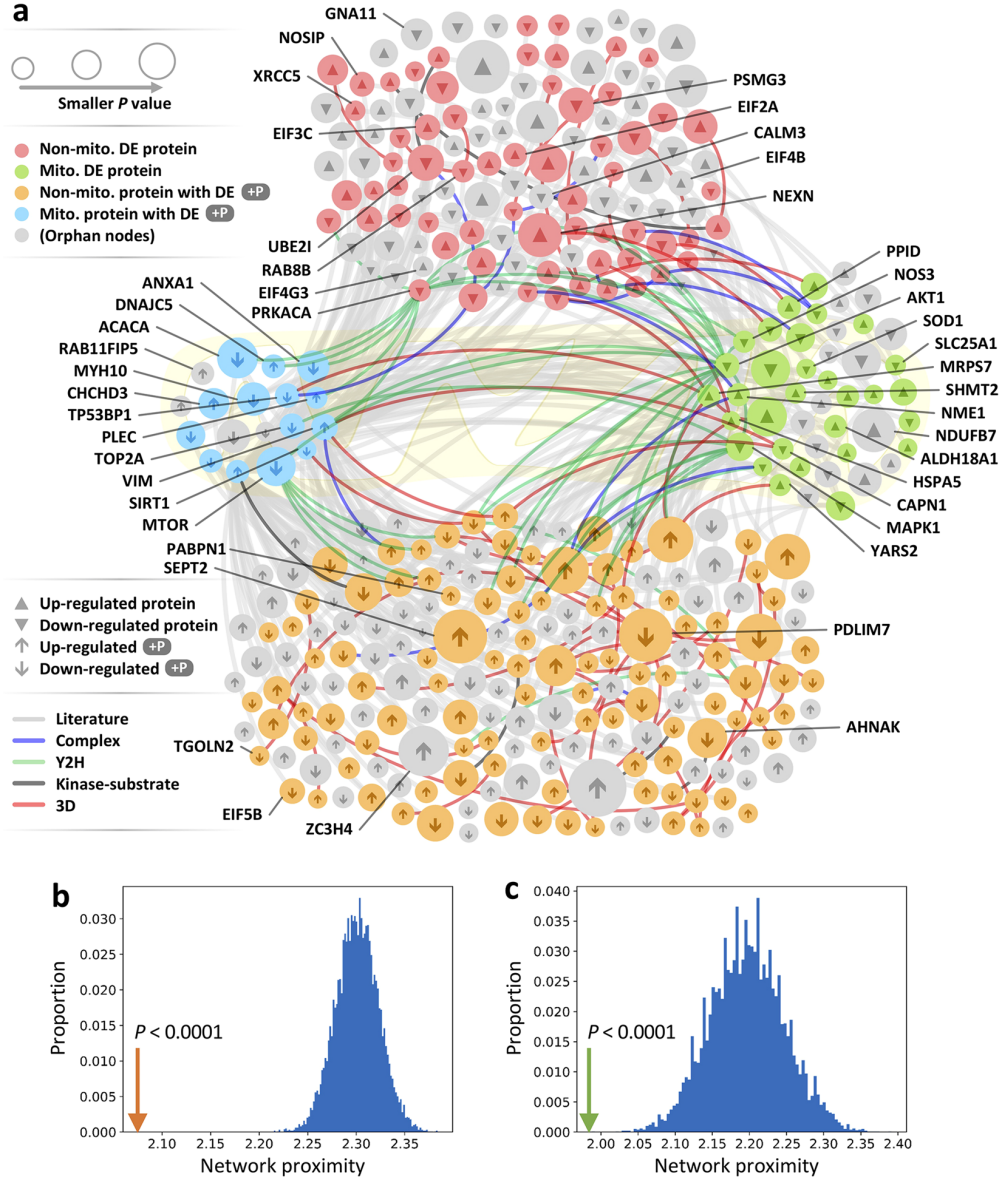
An integrative human interactome network analysis in PAH. (**a**) The human interactome network analysis shows that differentially expressed proteins and phosphorylated proteins share many neighbors among mitochondrial proteins and mitochondrial phosphorylated proteins despite little overlap (Fig. 1). Protein-protein interaction (PPI) lines are labeled by types of experimental evidence (color key of lines at the left of figure) and serve as basis for constructing the network (see Methods). 3D: three-dimensional and Y2H: Yeast Two-Hybrid. Color-code circles at the left identify whether data is from proteome [Non-mito. DE protein], mitochondrial proteome [Mito. DE protein], phosphoproteome [Non-mito. protein with DE + P], or mitochondrial phosphoproteome [Mito. protein with DE + P]. Node size is proportional to *p*-value of differential expression analysis. Names of proteins are in Supplementary Table S1, phosphorylated proteins in Supplementary Table S7, mitochondrial proteins in Supplementary Table S13, and mitochondrial phosphorylated proteins in Supplementary Table S15. (**b**) Differentially expressed proteins had significant network proximity with differentially expressed phosphorylated proteins in the human interactome network (orange arrow), although they did not have significant overlap at the protein level. Blue bars indicate the proximity distribution from a permutation test repeated 10,000 times using randomly selected proteins that preserve the degree distribution. Network proximity was calculated using the “Shortest” method. (**c**) Mitochondrial differentially expressed proteins had significant network proximity with mitochondrial differentially expressed phosphorylated proteins in the human interactome (green arrow).

Human interactome network analyses play crucial roles in drug target discovery and in identifying pathobiological pathways in multiple complex diseases^33,34^. To build a comprehensive human protein-protein interactome, we assembled data from 18 bioinformatics and systems biology databases with multiple experimental pieces of evidence (see Methods). We focused on experimentally validated protein-protein interactions (PPIs), and the resulting human interactome includes 351,444 PPIs connecting 17,706 unique proteins^33,34^. Subnetworks illustrated the full PPIs, highlighting the PAH disease module formed by differentially expressed proteins (*P* < 0.05, permutation test) and proteins with differentially phosphorylated sites (*P* < 0.05, permutation test) (Fig. 3a). The interactome network analyses show that differentially expressed proteins have significant network proximity (see Methods) with differentially expressed phosphorylated proteins in the human interactome network (*P* < 0.0001, permutation test, Fig. 3b), despite non-significant gene/protein overlap (*P* > 0.05, Fisher’s exact test). In addition, mitochondrial differentially expressed proteins had significant network proximity with mitochondrial differentially expressed phosphorylated proteins in the human interactome as well (*P* < 0.0001, permutation test, Fig. 3c). However, traditional protein-overlap analysis shows no overlap between mitochondrial differentially expressed proteins and mitochondrial differentially expressed phosphorylated proteins (*P* > 0.05, Fisher’s exact test, Fig. 1). For example, PPIs from three-dimensional (3D) protein structures illustrated interactions between mitochondrial protein calpain-1 catalytic subunit (CAPN1) and mitochondrial phosphorylated protein VIM, and between mitochondrial protein HSPA5 and mitochondrial phosphorylated protein TP53BP1. Furthermore, binary PPIs tested by high-throughput yeast-two-hybrid (Y2H) systems illustrated interactions between mitochondrial protein MAPK1 and mitochondrial phosphorylated protein TOP2A, and among mitochondrial protein AKT1 and mitochondrial phosphorylated proteins VIM and NAD-dependent protein deacetylase SIRT1 (Fig. 3a). Thus, human interactome network analyses highlight the power to investigate pathobiological pathways related to PAH as compared to traditional bioinformatics analysis (gene/protein overlap).

### Differential metabolite analysis in PAH patients

**Figure 4:**
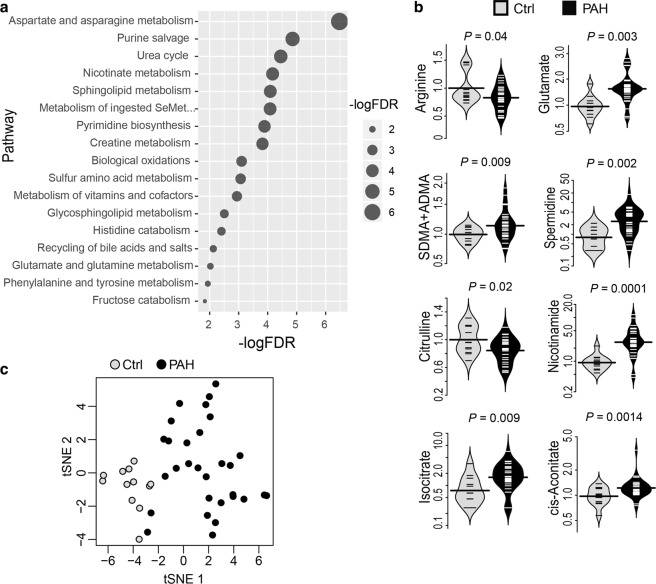
Nontargeted metabolomics analysis in PAH. (**a**) Pathway enrichment analysis revealed significant changes in differential metabolites between PAH and controls (false discovery rate [FDR]). (**b**) Relative abundances (Beanplots) of arginine, glutamate, symmetric dimethylarginine (SDMA) + asymmetric dimethylarginine (ADMA), spermidine, citrulline, nicotinamide, isocitrate, and cis-aconitate in plasma were significantly different between PAH (*n* = 30) and healthy controls (*n* = 12). Beanplots were prepared using R 3.5.1. (**c**) t-Distributed Stochastic Neighbor Embedding (t-SNE) plot showed clear separation of PAH group from healthy controls. t-SNE plot (2D projections) was generated based on normalized metabolomics profiles from PAH and healthy controls using the package Rtsne (version R 3.5.1).

To confirm that molecules and pathways associated with PAH in PAEC were relevant in PAH *in vivo*, we performed non-targeted metabolomics and targeted high-performance liquid chromatography of metabolites in plasma from PAH patients (*n* = 30) and healthy controls (*n* = 12) from a cohort previously described^27,35,36^ (Supplementary Table S19). Using non-targeted metabolomics analysis, we identified 1,583 distinct named metabolites and 374 unnamed metabolites in the plasma samples (Supplementary Table S20). A total of 339 metabolites were significantly (*P* < 0.05) altered in plasma from PAH patients. Of these, 198 metabolites were higher and 141 were lower in PAH as compared with healthy controls. In addition, changes in 170 metabolites (113 up, 57 down) approached significance (0.05 < *P* < 0.10) (Supplementary Table S20). Pathway enrichment analysis revealed significant changes in 17 biochemical pathways (*P* < 0.05, fold change > 1) relative to the overall change in PAH subjects, which included aspartate and asparagine metabolism, purine salvage, urea cycle, nicotinate metabolism and sphingolipid metabolism (Fig. 4a). Similar to previously reported metabolic abnormalities described in PAH^18,19^, we found significantly increased levels of glutamate, isocitrate, *cis*-aconitate, and purine metabolites including fMet, guanosine, adenosine, inosine, xanthosine, and hypoxanthine. Metabolomic analyses also showed significantly reduced glycine, arginine, and citrulline levels in PAH (all *P* < 0.05) (Fig. 4b, Supplementary Figs. S1 and S2, Supplementary Table S20). A total of 631 metabolites (excluding lipid metabolites) were utilized for t-Distributed Stochastic Neighbor Embedding (t-SNE) analysis. t-SNE analysis showed clear separation of the PAH group from healthy controls (Fig. 4c). We next sought to perform functional and integrative analysis to further understand the mechanism underlying metabolic abnormalities.

### Integrative analysis of mitochondrial proteomics and metabolomics

**Figure 5:**
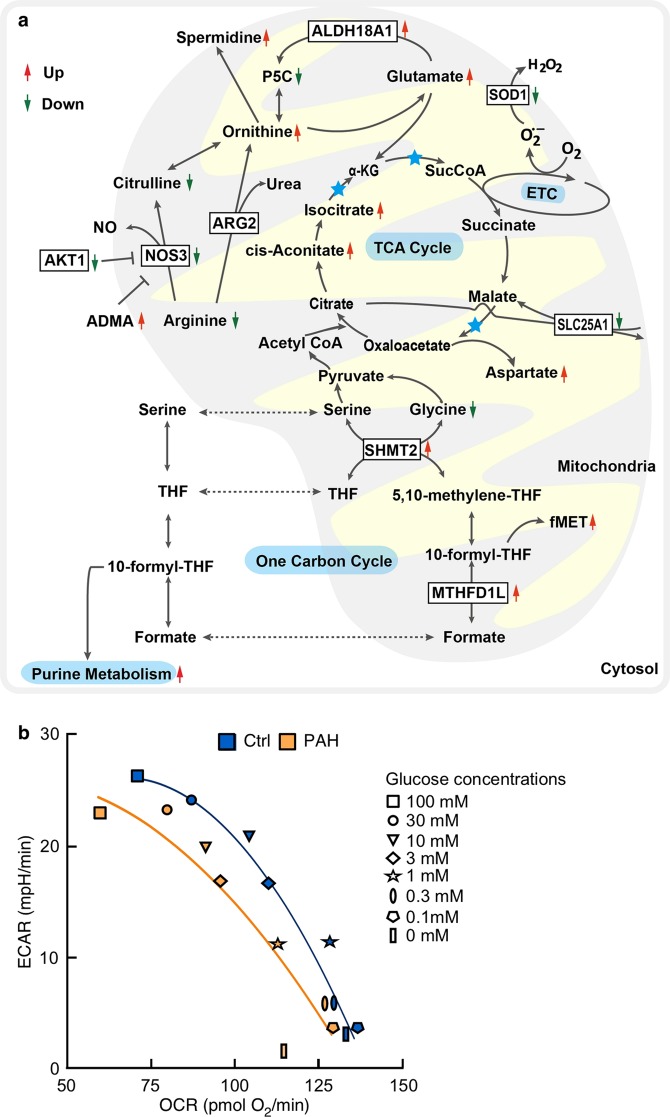
Dysregulated biological pathways and mitochondrial respiration in PAH. (**a**) Integrative analysis of proteomics and metabolomics reveals metabolic changes in PAH. Red arrow denotes elevated in PAH, and green arrow denotes decreased in PAH. Differentially expressed mitochondrial proteins by proteomics framed with black. Blue star, NAD^+^ is reduced to NADH. ADMA, asymmetric dimethylarginine; αKG, alpha-ketoglutarate; AKT1, RAC-alpha serine/threonine-protein kinase; ALDH18A1, delta-1-pyrroline-5-carboxylate synthase; ARG2, arginase 2; ETC, electron transport chain; fMet, N-formylmethionine; MTHFD1L, monofunctional C1-tetrahydrofolate (THF) synthase; NO, nitric oxide; NOS3, endothelial nitric oxide synthase; P5C, Δ1-pyrroline-5-carboxylate; SHMT2, serine hydroxymethyltransferase; SLC25A1, tricarboxylate transport protein; SOD1, superoxide dismutase [Cu-Zn]; TCA cycle, tricarboxylic acid cycle, THF, tetrahydrofolate. (**b**) Extracellular acidification rate (ECAR) vs. oxygen consumption rate (OCR) in PAH PAEC and healthy control PAEC. Mean of measurements are shown following addition of glucose at specified doses. PAH PAEC (*n* = 10) had a significant shift of the curve compared to control PAEC (*n* = 9) (*P* = 0.04).

To comprehensively assess metabolic changes in PAH cells as compared to control cells, we built an integrative metabolite-enzyme network analysis (see Methods) by assembling data from three commonly used metabolism databases: Kyoto Encyclopedia of Genes and Genomes (KEGG)^37^, Recon3D^38^, and human metabolic atlas^39^. Via an integrative network analysis of the metabolite-enzyme network and differentially expressed proteins derived from mitochondrial proteomics in PAEC, we identified several dysregulated pathways including SHMT2, MTHFD1L, NOS3, AKT1, SLC25A1, ALDH18A1, and superoxide dismutase [Cu-Zn] (SOD1) (Fig. 5a, Supplementary Fig. S3a). Key pathways identified through proteomic and metabolomic analyses are described in greater details with their relevance to PAH. To assess mitochondrial function in PAH PAEC, oxygen consumption rate (OCR) and extracellular acidification rate (ECAR) were measured using a Seahorse XF24 analyzer. We found that basal respiration in PAH PAEC (*n* = 3) was significantly lower than in control PAEC (*n* = 5) (basal OCR pmol O_2_/min, Controls 72 ± 4, PAH 44 ± 14, *P* = 0.03, Wilcoxon test). PAH PAEC had less oxygen consumption than control PAEC for any glucose dose provided to cells. As compared to control PAEC (*n* = 9), the curve of PAH PAEC (*n* = 10) was significantly shifted to the left (*P* = 0.04) (Fig. 5b). These results indicate functional biologic validation of changes in metabolic pathways of PAH PAEC compared to control cells, with PAH cells preferring glycolytic pathways and less oxidative metabolism, i.e., the Warburg phenomenon.

#### Reduced antioxidant response

SOD1, which binds copper and zinc ions, is a ubiquitous enzyme with an essential function in protecting aerobic cells against oxidative stress and is mainly expressed in the matrix of the mitochondria. SOD1 acts as a homodimer to convert superoxide to oxygen and hydrogen peroxide. Previously we and others identified increased oxidative stress and decreased SOD activity in PAH lungs and PAEC^1,5,40–42^. Here, significantly reduced expression of SOD1 detected by proteomics (*P* = 0.04) (Fig. 5a, Supplementary Table S14) was confirmed by Western blot analyses (Controls 1 ± 0.1, *n* = 7, PAH 0.7 ± 0.1, *n* = 7, *P* = 0.01, one-tailed *t*-test) (Supplementary Fig. S3b). To evaluate oxidative stress in PAH PAEC, menadione, which undergoes redox cycling to produce ROX via NADPH cytochrome P450 reductase and mitochondrial complex I (NADH-Ubiquinone oxidoreductase), was used to assess the capacity of PAEC to produce ROS. Baseline unstimulated ROS in quiescent control (*n* = 5) and PAH PAEC (*n* = 4) were similar (CellROX median fluorescence intensity Controls, 2453 ± 541; PAH 2051 ± 731, *P* = 0.6), but menadione led to a subset of cells in both PAH and controls that exhibited high CellROX staining (% CellROX^hi^, Controls 29 ± 13; PAH 28 ± 14, *P* = 0.9). Within the CellROX^hi^ subset, the ROS level generated by PAH PAEC exposed to menadione was significantly higher than in control cells (CellROX Median Fluorescence Intensity menadione/baseline, Controls 0.90 ± 0.05; PAH 1.06 ± 0.06, *P* = 0.04; one-tailed *t*-test), indicating greater capacity for ROS generation in PAH PAEC under conditions of redox stress. In addition, mitochondrial protein MGST1, which protects against oxidative stress and regulates mitochondrial metabolism^43^, was downregulated in PAH (*P* = 0.01) (Supplementary Tables S14 and S17). Glutathione peroxidases (GPX) are additional antioxidants that play a major role in the protection against oxidative stress by detoxification of hydrogen peroxide. Network analysis showed that two isoforms of GPX were lower in PAH (GPX1, *P* = 0.06; GPX4, *P* = 0.11) (Supplementary Table S1), and STRING analysis reveals that they are partners of SOD1. Taken together, network analysis offers potential evidence that increased oxidative stress in PAH is due to loss of antioxidant response (Fig. 5a).

#### Accelerated one carbon metabolism

One carbon metabolism in mitochondria is essential for the biosynthesis required for cell proliferation and pivotal for redox balance during hypoxia^44,45^. Proteomic studies revealed two key enzymes in one carbon pathway [SHMT2 (*P* = 0.036) and MTHFD1L (*P* = 0.04)], both of which affect organonitrogen compound biosynthetic and metabolic process pathways, were significantly elevated in PAH (Supplementary Fig. S3a, Supplementary Tables S14 and S17). Metabolomics data were consistent with proteomics data (Fig. 4, Supplementary Fig. S1). SHMT2 is a mitochondrial protein that catalyzes serine and glycine to tetrahydrofolate (THF) and 5,10-methylene-THF. Interestingly, global metabolomics of PAH revealed lower plasma glycine levels (Supplementary Fig. S1) supporting higher enzyme activity of SHMT2. MTHFD1L catalyzes 10-formyl-THF, an important precursor in purine and N-formylmethionine (fMet) synthesis, to formate. Global plasma metabolomics identified an increase in purine metabolites in PAH, including fMet (*P* = 0.02), guanosine (*P* = 0.001), adenosine (*P* = 0.0001), inosine (*P* = 0.005), xanthosine (*P* = 0.04), and hypoxanthine (*P* = 0.002) (Supplementary Fig. S1), supporting the hyperproliferative phenotype of PAH PAEC as previously reported^1,3^. Integrative network analyses of proteomics and metabolomics indicate that one carbon pathway is dysregulated in PAH (Fig. 5a).

#### Downregulated nitric oxide and arginine pathways

The hallmark of endothelial vascular dysfunction in PAH is the impaired production of NO by NOS3. Underlying mechanisms include loss of NOS3, inactivation of NOS3, and/or decreased NOS3 substrate arginine bioavailability due to increased arginases^2,4,10,46^. Here, NOS3 protein and AKT1, which positively regulates NOS activity via phosphorylation of NOS3^47^, were significantly lower in PAH PAEC (Supplementary Fig. S3a, Supplementary Table S14). Other proteins in the arginine/NO pathway were also differentially expressed, such as NOS-interacting protein (NOSIP), cAMP-dependent protein kinase catalytic subunit alpha (PRKACA), and CALM3 (Supplementary Tables 2 and 3). The plasma metabolome of PAH also shows that endogenous NOS inhibitors dimethylarginines (DMA) (symmetric DMA + asymmetric DMA) and monomethylarginine (MMA) were significantly increased in PAH (Fig. 4b, Supplementary Fig. S2). Furthermore, arginine and citrulline levels were significantly lower in plasma of PAH patients (Fig. 4b, Supplementary Fig. S2). Arginase, a critical enzyme in the urea cycle, converts arginine to ornithine and urea and plays a regulatory role in NO synthesis by modulating the availability of arginine for NOS^48^. Because arginases are intracellular enzymes that appear in the circulation only after cell damage or death, arginine-to-ornithine ratio has been suggested as a better assessment of total-body arginase activity^49^. The arginine-to-ornithine ratio was lower in plasma from PAH patients compared with controls (*P* = 0.04) (Supplementary Fig. S2), indicating substrate limitation for NOS in PAH *in vivo*^2,46^. Collectively, the proteome and metabolome of PAH confirms that the NOS3 pathway is impaired (Fig. 5a).

#### Abnormal tricarboxylic acid (TCA) cycle flux

TCA cycle has a primary role in oxidation of substrate for energy production (ATP) but also serves critical biosynthetic functions in which intermediates enter and leave the cycle to regulate cell metabolism and signal transduction^50^. SLC25A1, a mitochondrial citrate carrier, exports citrate from mitochondria to cytoplasm (Supplementary Table S17)^51^. SLC25A1 mutations that inactivate the citrate export pathway lead to severe mitochondrial dysfunction^51^. Proteomic study of PAEC showed lower levels of SLC25A1 in PAH (*P* = 0.03) (Supplementary Fig. S3a, Supplementary Table S14) that were confirmed by Western blot analyses (Controls 1 ± 0.1, *n* = 5, PAH 0.4 ± 0.1, *n* = 4, *P* = 0.01, one-tailed *t*-test) (Supplementary Fig. S3b), suggesting that the concentration of mitochondrial citrate may be higher than cytosolic citrate. The plasma metabolome showed higher isocitrate (*P* = 0.009) and *cis*-aconitate (*P* = 0.014) in PAH (Fig. 4b). In addition, citrate was previously reported to be increased in PAH lungs^21^. The findings suggest that decreased SLC25A1 protein influences the TCA cycle flux in PAH (Fig. 5a).

#### Alteration of glutamate metabolism in PAH

ALDH18A1 catalyzes glutamate to Δ1-pyrroline-5-carboxylate (P5C), a major step in the biosynthesis of proline, ornithine, and arginine^52,53^. Proteomics showed higher levels of ALDH18A1 (*P* = 0.02) (Supplementary Fig. S3a, Supplementary Table S14). Consistent with our result, others have found increased expression of ALDH18A1 in PAH lungs^21^. High levels of plasma glutamate (*P* = 0.003), a substrate of ALDH18A1, and spermidine (*P* = 0.002), downstream of ornithine, were found in PAH (Fig. 4b). The upregulated ALDH18A1 and consequent elevated spermidine in PAH (Figs. 4b and 5a) is similar to findings in rapidly growing malignant cells as predicted by IPA (Supplementary Table S6).

#### Abnormal mitochondrial phosphoproteins related to fatty acid metabolic pathway, cristae morphology, and deacetylase activity of PAH PAEC

Acetyl-CoA carboxylase 1 (ACACA), mitochondrial contact site and cristae organizing system (MICOS) complex subunit MIC19 (CHCHD3), and SIRT1 were some of the differentially expressed mitochondrial phosphoproteins (Supplementary Table S16).

An imbalance between glycolysis, glucose oxidation, and fatty acid oxidation has been reported in PAH^21,54^. Mitochondrial ACACA, a downstream target of AMP-activated protein kinase (AMPK), catalyzes a rate-limiting reaction in the biogenesis of long-chain fatty acids. Recent reports show that knockdown of ACACA by siRNA limits fatty acid supply to TCA cycle and induces apoptosis in cancer cells. Phosphorylation of ACACA via AMPK leads to inactivation and changes in metabolism of cancer cells^55,56^. Thus, the low phosphorylation state of ACACA in PAH (*P* = 0.001)(Supplementary Table S16) may enhance catalytic function and increase fatty acid metabolism in PAH^57^. Consistent with lower phosphorylated ACACA, other proteins involved in fatty acid metabolism, e.g., ACAT2 and ECI1, were also differentially expressed in PAH. ACAT2 protein levels were significantly decreased in PAH (*P* = 0.001) (Supplementary Table S14), while ECI1 protein levels were significantly increased in PAH (*P* = 0.004) (Supplementary Table S14), indicating abnormal fatty acid metabolic pathways in PAH.

CHCHD3, an inner mitochondrial membrane protein, plays an important role in the maintenance of the mitochondrial contact site and cristae-organizing system (MICOS) complex stability and mitochondrial cristae morphology^58^. Mitochondrial cristae are the site of oxidative phosphorylation (OXPHOS) and modulators of mitochondrial bioenergetics^59^. Alteration of phosphorylation of CHCHD3 (*P* = 0.007) could lead to reductions in oxygen consumption and ATP production and affect cell growth, cell death, and survival (Supplementary Tables 12 and 16)^58^. Two functional partners of CHCHD3, mitochondrial-localized MICOS complex subunit MIC27 (APOOL) and coiled-coil-helix-coiled-coil-helix domain-containing protein 2 (CHCHD2) predicted by STRING analyses, were identified by proteomics (Supplementary Table S13). Protein levels of CHCHD2 required for MICOS complex at the cristae junction tended to be increased in PAH (*P* = 0.10) (Supplementary Table S13)^60^. Downregulated APOOL protein levels (*P* = 0.07) (Supplementary Table S13) would cause major alterations in cristae morphology and impair mitochondrial respiration^61^.

Mitochondrial phosphor-SIRT1, one of the seven sirtuins linked to longevity in mammals, had significantly reduced phosphorylation in PAH (*P* = 0.04) (Supplementary Table S16). Sirtuins mediate NAD+ -dependent deacetylation of targets such as AKT signaling and peroxisome proliferator-activated receptor gamma coactivator 1-alpha (PGC-1α) (Supplementary Tables S10–S12, and S18)^62,63^. PPI between SIRT1 and AKT1 was identified by high-throughput Y2H systems (Fig. 3a). Sirtuin signaling pathway-related proteins, such as NDUFB7 (*P* = 0.0009), peptidyl-prolyl *cis-trans* isomerase D (PPID) (*P* = 0.02), and X-ray repair cross-complementing protein 5 (XRCC5) (*P* = 0.04) were significantly upregulated in proteomic analysis (Supplementary Tables 2, 3 and 14). Metabolomics also showed that nicotinamide, as part of the coenzyme nicotinamide adenine dinucleotide (NADH / NAD+), was increased in PAH (*P* < 0.0001) (Figs. 4b and 5a). These integrative network analyses support that abnormal phosphorylation of SIRT1 might cause a decline in its deacetylase activity, leading to metabolic redox perturbations in PAH (Supplementary Table S18)^64^.

## Conclusion

In this study, 170 of 2,556 proteins and 240 of 3,609 phosphopeptides were significantly different between PAH PAEC and control PAEC. Among the proteins encoded by mtDNA or transcribed from nuclear DNA and imported to the mitochondria, 670 mitochondrial proteins and 366 mitochondrial phosphopeptides were identified, including 45 mitochondrial differentially expressed proteins and 18 mitochondrial differentially expressed phosphopeptides. Many of these proteins were previously unknown and unsuspected to be altered in PAH. An integrative network analysis of multi-omics data in PAEC and plasma uncovered dysregulated pathways particularly related to mitochondria, e.g., accelerated one carbon metabolism, abnormal TCA cycle flux and glutamate metabolism, dysfunctional arginine and NO pathways, and enhanced oxidative stress. Functional studies in PAH PAEC confirmed decreases in mitochondrial oxygen consumption and increases in oxidative stress. Overall, the complementary and synergistic integrative analyses of proteomics and metabolomics provide an unparalleled opportunity to achieve a broad understanding of mechanisms underlying the disease and identify new targets for future therapeutics.

## Methods

### Pulmonary arterial endothelial cell cultures

Human primary pulmonary arterial endothelial cells (PAEC) were collected either from unused explanted control donor lungs or explanted from PAH patients undergoing lung transplantation who provided informed written consent or under an IRB exempt protocol. The study was approved by the Institutional Review Board of the Cleveland Clinic. All methods were performed in accordance with the relevant guidelines and regulations.

PAEC from fifteen explanted PAH lungs or ten donor lungs not used for transplantation were harvested and cultured as previously described^4,10,11^. Four additional control PAEC were purchased from Lonza (Walkersville, MD). Cells were passaged at 70–80% confluency, and primary cultures of passages 6–7 used in experiments. Purity of the cultured PAEC were evaluated with flow cytometry. The purity of each PAEC was assessed with CD31; the mean purity of PAEC was 98.5% and individual cultures were all above 96.8% (Supplementary Table S21)^11^. There was no difference between control PAEC commercially purchased or harvested at Cleveland Clinic; they were identical in expression patterns for proteins (Supplementary Fig. S4, Supplementary Table S21).

### Mass spectrometry analysis

For global proteomics, a 50 μg aliquot of protein from each PAEC sample was subjected to in-gel digestion in which the whole gel lane was divided into 13 areas, and the gel pieces were washed/destained in 50% ethanol, 5% acetic acid, dehydrated in acetonitrile, and then prepared as previously described^26,65^. MaxQuant V1.5.2.8 with the search engine Andromeda integrated into MaxQuant software was used to analyze the data, and default settings were used as the parameters for the Orbitrap instrument. The database used to search the MS/MS spectra was the Uniprot human protein database containing 85,299 protein sequences. CID spectra collected in the experiment were used for peptide sequencing and protein identification, and full scans were used for peptide precursor intensity calculations. For targeted proteomics, a parallel reaction monitoring (PRM) experiment was performed on a Thermo Scientific Fusion Lumos instrument. The peptides targeted in these analyses were chosen based on the presence in the initial data dependent analyses, unique to the protein of interest, and the shape of the chromatographic peaks^66^.

For global phosphoproteomic analysis, a 1.0 mg aliquot of protein from each PAEC sample was subjected to serine and threonine phosphorylation enrichment using Thermo Scientific™ Pierce™ TiO2 (Thermo Scientific™ Pierce™) and C18 clean-up (Fisher # PI88301) prior to LC-MS/MS analysis. The LC-MS/MS system was a Thermo Fisher Scientific LTQ-Obitrap Elite hybrid mass spectrometer. The data was acquired using the data dependent method described above. MaxQuant V1.5.2.8 with the search engine Andromeda integrated into MaxQuant software was used to analyze the data, and default settings were used as the parameters for the Orbitrap instrument with the addition of phosphorylation at S, T, and Y residues as a variable modification. Phosphorylated peptides containing one or more modification sties were considered. The site localization threshold of greater than 50% was used and this resulted in a majority of phosphorylation events occurring on Serine residues, 6,113 (84.3%), followed by Threonine, 1,065 (14.7%), with 74(1%) occurring on Tyrosine residues.

Label‐free quantitation (LFQ) for both the proteomics and phosphoproteomics by MaxQuant was used to determine intensity and normalize protein quantities. LFQ intensities are the output of the MaxLFQ algorithm^14^.

### Quantitative analysis of proteome and phosphoproteome

Patient characteristics were summarized with appropriate descriptive statistics. For the protein expression analysis, the variance-stabilizing transformation (VSN) was first performed to normalize the proteomics and phosphoproteomics data, respectively. Missing data were imputed using the nearest neighbor approach. The comparison between PAH and healthy controls was then performed using the moderated *t*-test (*limma* package in R). The results were controlled for multiple comparisons with the false discovery rate (FDR) approach, and proteins with significantly different expressions were identified with FDR < 0.05. All analyses were conducted using R-studio (Boston, MA).

### Oxygen consumption rate (OCR) and extracellular acidification rate (ECAR)

OCR and ECAR were measured using the Seahorse Extracellular Flux (XF24) Analyzer (Seahorse Bioscience Inc. North Billerica, MA) according to manufacturer’s protocol. Seahorse assay media (DMEM without glucose, l-glutamine, phenol red, sodium pyruvate, and sodium bicarbonate [Sigma-Aldrich] supplemented with either 1.08 g/l glucose, 1.85 g/l sodium chloride, 1 mM sodium pyruvate, and 15 mg/l phenol red [MitoStress Assay] or 1.85 g/l sodium chloride and 3 mg/l phenol red [glucose dose response assay]) was supplemented with 2 mM l-glutamine and the pH adjusted to 7.35 with sodium hydroxide. PAEC were plated at a density of 50,000 cells per well in MCDB107 growth media overnight, with 3 wells per plate left empty for background correction. Growth medium was removed, and cells were washed with the appropriate Seahorse assay medium three times. After the final wash, assay medium was added to each well at a final per-well volume of 500 μl. The plate was incubated in a 37 °C non-CO_2_ incubator for one hour. The plate was then transferred to the Seahorse XF24 Analyzer for analysis. For the basal oxygen measurement, OCR was measured in PAEC in assay medium containing glucose, with 5 replicates per cell. For the glucose dose response test, PAEC underwent basal measurement of extracellular acidification in glucose-free assay medium, followed by addition of glucose at 0 mM, 0.1 mM, 0.3 mM, 1 mM, 3 mM, 10 mM, 30 mM, or 100 mM, with 2–3 wells per glucose concentration. All measures were done 3 times in a 3–2–3-minute mix-wait-measure cycle. The area under the curve for OCR and ECAR was compared between PAH PAEC and control PAEC using the bootstrap method.

### Signaling pathway analyses of proteome and phosphoproteome

Canonical pathway analysis, upstream regulator analysis, and interaction network analysis of differentially expressed proteins as well as phosphoproteins were done with IPA (Qiagen, Redwood City). The search tool for the retrieval of interacting genes/proteins (STRING) (http://string-db.org) was used to predict functional partners and biological pathways.

### Construction of human protein-protein interactome

To build a comprehensive human interactome network, we integrated data from a total of 18 differential bioinformatics databases with multiple experimental evidences. Specifically, we used PPIs with six types of experimental evidence: (i) binary PPIs tested by systematic, high-throughput yeast-two-hybrid (Y2H) systems; (ii) binary PPIs from three-dimensional (3D) protein structure data; (iii) kinase-substrate interactions from literature-derived experimental data; (iv) protein signaling network from literature-derived low-throughput experiments, (v) protein complexes (approximately 56,000 PPIs) tested by an affinity purification-mass spectrometry assay, and (vi) literature-curated PPIs identified by affinity purification followed by mass spectrometry (AP-MS), Y2H, and by literature-derived experimental data. The genes have been mapped to Entrez ID and their official gene symbols based on GeneCards (http://www.genecards.org/). We removed all computationally predicted data, such as evolutionary analysis, gene co-expression network, and metabolic associations. In total, the resulting human interactome included 351,444 PPIs connecting 17,706 unique proteins. More detailed descriptions are provided in our recent studies^33,34^.

### Network proximity analysis

To inspect the network relationship between differentially expressed proteins and differentially expressed phosphorylated proteins, we performed network proximity analysis. Specifically, network proximity quantifies the average shortest path length between two different protein sets in the human interactome. Given *A* and *B*, the set of proteins for *A* (e.g., differentially expressed proteins) and *B* (e.g., differentially phosphorylated proteins), and *d*_*AB*_, the shortest path length between nodes *a* and *b*, we define the network proximity measure as follows:

1 $$\langle {d}_{AB}^{s}\rangle =\frac{1}{\Vert {\rm{A}}\Vert \times \Vert {\rm{B}}\Vert }\sum _{a\in A,\,b\in B}\,d(a,b)$$

To evaluate the significance of the network distance between two protein sets, we built a reference distance distribution corresponding to the expected distance between two randomly selected groups of proteins with the same size and degree (connectivity) distribution as the original protein set. We repeated this procedure 10,000 times. The mean $\bar{d}$ and standard deviation (*σ*_*d*_) were used to calculate a z-score (*z*_*d*_) by converting an observed (non-Euclidean) distance to a normalized distance. We computed *P*-value by 10,000 permutation tests. More details of network proximity analysis are given in our previous studies^33,34^.

### Metabolomic analysis

Nontargeted metabolomic analysis was performed on plasma samples collected from 30 PAH and 12 healthy controls (Supplementary Table S19) by Metabolon (Durham, NC) as previously described^67^.

### Metabolite-enzyme network analyses

We built a comprehensive metabolite-enzyme network by assembling data from three commonly used metabolism databases: KEGG^37^, Recon3D^38^, and human metabolic atlas^39^. We then mapped the differentially expressed proteins (enzymes) and significant metabolites into the metabolite-enzyme network to identify the dysregulated metabolism pathways in PAH.

## Supplementary information

Supplementary Information

Supplementary Table S1

Supplementary Table S7

Supplementary Table S13

Supplementary Table S15

Supplementary Table S20

## Data Availability

All data associated with this study are available in the main text or the supplementary materials.

## References

[1] Xu W, Erzurum SC (2011). Endothelial cell energy metabolism, proliferation, and apoptosis in pulmonary hypertension. In American Physiological Society. Compr Physiol.

[2] Xu W (2004). Increased arginase II and decreased NO synthesis in endothelial cells of patients with pulmonary arterial hypertension. Faseb J.

[3] Masri FA (2007). Hyperproliferative apoptosis-resistant endothelial cells in idiopathic pulmonary arterial hypertension. Am J Physiol Lung Cell Mol Physiol.

[4] Xu W (2007). Alterations of cellular bioenergetics in pulmonary artery endothelial cells. Proc Natl Acad Sci USA.

[5] Fijalkowska I (2010). Hypoxia inducible-factor1alpha regulates the metabolic shift of pulmonary hypertensive endothelial cells. Am J Pathol.

[6] Kaneko FT (1998). Biochemical reaction products of nitric oxide as quantitative markers of primary pulmonary hypertension. Am J Respir Crit Care Med.

[7] Fagan KA, McMurtry I, Rodman DM (2000). Nitric oxide synthase in pulmonary hypertension: lessons from knockout mice. Physiol Res.

[8] Machado RF (2004). Nitric oxide and pulmonary arterial pressures in pulmonary hypertension. Free Radic Biol Med.

[9] Girgis RE (2005). Decreased exhaled nitric oxide in pulmonary arterial hypertension: response to bosentan therapy. Am J Respir Crit Care Med.

[10] Ghosh S (2016). Phosphorylation inactivation of endothelial nitric oxide synthesis in pulmonary arterial hypertension. Am J Physiol Lung Cell Mol Physiol.

[11] Comhair SA (2012). Human primary lung endothelial cells in culture. Am J Respir Cell Mol Biol.

[12] Kim J (2013). An endothelial apelin-FGF link mediated by miR-424 and miR-503 is disrupted in pulmonary arterial hypertension. Nat Med.

[13] Sofer A (2018). Therapeutic Engagement of the Histone Deacetylase IIA-Myocyte Enhancer Factor 2 Axis Improves Experimental Pulmonary Hypertension. Am J Respir Crit Care Med.

[14] Cox J (2014). Accurate proteome-wide label-free quantification by delayed normalization and maximal peptide ratio extraction, termed MaxLFQ. Mol Cell Proteomics.

[15] Rhodes CJ (2017). Plasma proteome analysis in patients with pulmonary arterial hypertension: an observational cohort study. Lancet Respir Med.

[16] Abdul-Salam VB (2010). Proteomic analysis of lung tissues from patients with pulmonary arterial hypertension. Circulation.

[17] Yao C (2015). Protein Expression by Human Pulmonary Artery Smooth Muscle Cells Containing a BMPR2 Mutation and the Action of ET-1 as Determined by Proteomic Mass Spectrometry. Int J Mass Spectrom.

[18] Fessel JP (2012). Metabolomic analysis of bone morphogenetic protein receptor type 2 mutations in human pulmonary endothelium reveals widespread metabolic reprogramming. Pulm Circ.

[19] Bertero T (2016). Vascular stiffness mechanoactivates YAP/TAZ-dependent glutaminolysis to drive pulmonary hypertension. J Clin Invest.

[20] Eyries M (2014). EIF2AK4 mutations cause pulmonary veno-occlusive disease, a recessive form of pulmonary hypertension. Nat Genet.

[21] Zhao Y (2014). Metabolomic heterogeneity of pulmonary arterial hypertension. PLoS One.

[22] Zhong JC (2007). Apelin modulates aortic vascular tone via endothelial nitric oxide synthase phosphorylation pathway in diabetic mice. Cardiovasc Res.

[23] Hadinnapola C (2017). Phenotypic Characterization of EIF2AK4 Mutation Carriers in a Large Cohort of Patients Diagnosed Clinically With Pulmonary Arterial Hypertension. Circulation.

[24] Montani D (2017). Clinical phenotypes and outcomes of heritable and sporadic pulmonary veno-occlusive disease: a population-based study. Lancet Respir Med.

[25] Kang Y (2013). Apelin-APJ signaling is a critical regulator of endothelial MEF2 activation in cardiovascular development. Circ Res.

[26] Cheong HI (2016). Hypoxia sensing through beta-adrenergic receptors. JCI Insight.

[27] Farha, S. *et al*. Pulmonary arterial hypertension treatment with carvedilol for heart failure: a randomized controlled trial. *JCI Insight* **2** (2017). 10.1172/jci.insight.95240 PMC5621927 28814664

[28] Jiang X, Feng S, Chen Y, Feng Y, Deng H (2016). Proteomic analysis of mTOR inhibition-mediated phosphorylation changes in ribosomal proteins and eukaryotic translation initiation factors. Protein Cell.

[29] Gavish-Izakson M (2018). Nuclear poly(A)-binding protein 1 is an ATM target and essential for DNA double-strand break repair. Nucleic Acids Res.

[30] Jiang R (2019). The emerging roles of a novel CCCH-type zinc finger protein, ZC3H4, in silica-induced epithelial to mesenchymal transition. Toxicol Lett.

[31] Bonnet S (2006). An abnormal mitochondrial-hypoxia inducible factor-1alpha-Kv channel pathway disrupts oxygen sensing and triggers pulmonary arterial hypertension in fawn hooded rats: similarities to human pulmonary arterial hypertension. Circulation.

[32] Kraja AT (2019). Associations of Mitochondrial and Nuclear Mitochondrial Variants and Genes with Seven Metabolic Traits. Am J Hum Genet.

[33] Cheng F (2018). Network-based approach to prediction and population-based validation of in silico drug repurposing. Nat Commun.

[34] Cheng F, Kovacs IA, Barabasi AL (2019). Network-based prediction of drug combinations. Nat Commun.

[35] Cheong HI (2018). Endothelial Phenotype Evoked by Low Dose Carvedilol in Pulmonary Hypertension. Front Cardiovasc Med.

[36] Saygin D (2017). Metabolic and Functional Evaluation of the Heart and Lungs in Pulmonary Hypertension by Gated 2-[18F]-Fluoro-2-deoxy-D-glucose Positron Emission Tomography. Pulm Circ.

[37] Kanehisa M, Sato Y, Furumichi M, Morishima K, Tanabe M (2019). New approach for understanding genome variations in KEGG. Nucleic Acids Res.

[38] Brunk E (2018). Recon3D enables a three-dimensional view of gene variation in human metabolism. Nat Biotechnol.

[39] Pornputtapong N, Nookaew I, Nielsen J (2015). Human metabolic atlas: an online resource for human metabolism. Database (Oxford).

[40] Masri FA (2008). Deficiency of Lung Antioxidants in Idiopathic Pulmonary Arterial Hypertension. Clinical and Translational Science.

[41] Grobe AC (2006). Increased oxidative stress in lambs with increased pulmonary blood flow and pulmonary hypertension: role of NADPH oxidase and endothelial NO synthase. Am J Physiol Lung Cell Mol Physiol.

[42] Xu, W., Erzurum, S. C. Airways inflammation and reactive oxygen/nitrogen species in pulmonary hypertension. In *Oxidative Stress: Clinical and Biomedical Implications*. Nova Science Publishers, Inc., pp. 259–276 (2007).

[43] Brautigam L (2018). MGST1, a GSH transferase/peroxidase essential for development and hematopoietic stem cell differentiation. Redox Biol.

[44] Jain M (2012). Metabolite profiling identifies a key role for glycine in rapid cancer cell proliferation. Science.

[45] Martinez-Reyes I, Chandel NS (2014). Mitochondrial one-carbon metabolism maintains redox balance during hypoxia. Cancer Discov.

[46] Kao CC (2015). Arginine metabolic endotypes in pulmonary arterial hypertension. Pulm Circ.

[47] Schleicher M (2009). The Akt1-eNOS axis illustrates the specificity of kinase-substrate relationships *in vivo*. Sci Signal.

[48] Ignarro LJ (2001). Role of the arginine-nitric oxide pathway in the regulation of vascular smooth muscle cell proliferation. Proc Natl Acad Sci USA.

[49] Morris CR (2003). Arginine therapy: a new treatment for pulmonary hypertension in sickle cell disease?. Am J Respir Crit Care Med.

[50] Owen OE, Kalhan SC, Hanson RW (2002). The key role of anaplerosis and cataplerosis for citric acid cycle function. J Biol Chem.

[51] Nota B (2013). Deficiency in SLC25A1, encoding the mitochondrial citrate carrier, causes combined D-2- and L-2-hydroxyglutaric aciduria. Am J Hum Genet.

[52] Fischer-Zirnsak B (2015). Recurrent De Novo Mutations Affecting Residue Arg138 of Pyrroline-5-Carboxylate Synthase Cause a Progeroid Form of Autosomal-Dominant Cutis Laxa. Am J Hum Genet.

[53] Skidmore DL (2011). Further expansion of the phenotypic spectrum associated with mutations in ALDH18A1, encoding Delta(1)-pyrroline-5-carboxylate synthase (P5CS). Am J Med Genet A.

[54] Talati M, Hemnes A (2015). Fatty acid metabolism in pulmonary arterial hypertension: role in right ventricular dysfunction and hypertrophy. Pulm Circ.

[55] Brusselmans K, De Schrijver E, Verhoeven G, Swinnen JV (2005). RNA interference-mediated silencing of the acetyl-CoA-carboxylase-alpha gene induces growth inhibition and apoptosis of prostate cancer cells. Cancer Res.

[56] Chajes V, Cambot M, Moreau K, Lenoir GM, Joulin V (2006). Acetyl-CoA carboxylase alpha is essential to breast cancer cell survival. Cancer Res.

[57] Vazquez-Martin A (2013). Serine79-phosphorylated acetyl-CoA carboxylase, a downstream target of AMPK, localizes to the mitotic spindle poles and the cytokinesis furrow. Cell Cycle.

[58] Darshi M (2011). ChChd3, an inner mitochondrial membrane protein, is essential for maintaining crista integrity and mitochondrial function. J Biol Chem.

[59] Cogliati S, Enriquez JA, Scorrano L (2016). Mitochondrial Cristae: Where Beauty Meets Functionality. Trends Biochem Sci.

[60] Huang X (2018). CHCHD2 accumulates in distressed mitochondria and facilitates oligomerization of CHCHD10. Hum Mol Genet.

[61] Weber TA (2013). APOOL is a cardiolipin-binding constituent of the Mitofilin/MINOS protein complex determining cristae morphology in mammalian mitochondria. PLoS One.

[62] Pillai VB, Sundaresan NR, Gupta MP (2014). Regulation of Akt signaling by sirtuins: its implication in cardiac hypertrophy and aging. Circ Res.

[63] de Kreutzenberg SV (2010). Downregulation of the longevity-associated protein sirtuin 1 in insulin resistance and metabolic syndrome: potential biochemical mechanisms. Diabetes.

[64] Sasaki T (2008). Phosphorylation regulates SIRT1 function. PLoS One.

[65] Tirupula KC (2015). MAS C-Terminal Tail Interacting Proteins Identified by Mass Spectrometry- Based Proteomic Approach. PLoS One.

[66] Willard BB, Ruse CI, Keightley JA, Bond M, Kinter M (2003). Site-specific quantitation of protein nitration using liquid chromatography/tandem mass spectrometry. Anal Chem.

[67] Comhair SA (2015). Metabolomic Endotype of Asthma. J Immunol.

