# Supplementary material for: Integrative proteomics and phosphoproteomics in pulmonary arterial hypertension
Source: Sci Rep. 2019 Dec 9;9:18623. doi: 10.1038/s41598-019-55053-6 (PMC6901481; doi:10.1038/s41598-019-55053-6)
Supplement: Supplementary file 1 — Supplementary Information [file 41598_2019_55053_MOESM1_ESM.pdf]

Supplementary Information

Integrative proteomics and phosphoproteomics in pulmonary arterial hypertension

Weiling Xu[1*], Suzy A. A. Comhair[1], Ruoying Chen[1], Bo Hu[1], Yuan Hou[1], Yadi Zhou[1], Lori A. Mavrakis[1], Allison J. Janocha[1], Ling Li[1], Dongmei Zhang[1], Belinda B. Willard[1], Kewal Asosingh[1], Feixiong Cheng[1], Serpil C. Erzurum[1,2*]

[1]Lerner Research Institute, Cleveland Clinic, Cleveland, Ohio, United States of America.

[2] Respiratory Institute, Cleveland Clinic, Cleveland, Ohio, United States of America.

*Corresponding authors: xuw@ccf.org and erzurus@ccf.org

To whom correspondence should be addressed:

Serpil C. Erzurum, M.D.

Cleveland Clinic

9500 Euclid Avenue, NC22

Cleveland, Ohio 44195, USA.

Telephone: 216-445-6624

Fax: 216-444-3279

E-mail: erzurus@ccf.org

List of includes:

Supplementary Table S1. 2,556 proteins quantified from proteomics
Supplementary Table S2. 170 differentially expressed proteins in PAH PAEC compared with healthy control PAEC (all $P < 0.05$)
Supplementary Table S3. Canonical pathways of proteins differentially expressed in PAH PAEC
Supplementary Table S4. Causal networks of proteins differentially expressed in PAH PAEC
Supplementary Table S5. Biological functions of proteins differentially expressed in PAH PAEC
Supplementary Table S6. Top diseases and functions in networks of proteins differentially expressed in PAH PAEC
Supplementary Table S7. 3,609 peptides quantified from phosphoproteomics
Supplementary Table S8. 240 differentially expressed phosphopeptides in PAH PAEC compared with healthy control PAEC (all $P < 0.05$)
Supplementary Table S9. Canonical pathways of phosphoproteins differentially expressed in PAH PAEC
Supplementary Table S10. Causal networks of phosphoproteins differentially expressed in PAH PAEC
Supplementary Table S11. Biological functions of phosphoproteins differentially expressed in PAH PAEC
Supplementary Table S12. Top diseases and functions in networks of phosphoproteins differentially expressed in PAH PAEC
Supplementary Table S13. 670 Mitochondrial proteins identified in PAH PAEC and healthy control PAEC
Supplementary Table S14. 45 mitochondrial proteins differentially expressed in PAH PAEC compared with healthy control PAEC (all $P < 0.05$)
Supplementary Table S15. 366 Mitochondrial phosphopeptides identified in PAH PAEC and healthy control PAEC
Supplementary Table S16. 18 mitochondrial phosphopeptides differentially expressed between PAH PAEC and healthy control PAEC (all $P < 0.05$)
Supplementary Table S17. Biological functions of mitochondrial proteins differentially expressed in PAH PAEC
Supplementary Table S18. Top biological processes of mitochondrial phosphoproteins differentially expressed in PAH PAEC
Supplementary Table S19. Clinical features of study participants for metabolomics
Supplementary Table S20. Metabolite analysis in PAH versus healthy controls (PAHTCH Study)
Supplementary Table S21. Purity of PAEC used for all experiments as assessed with CD31

Supplementary Fig. S1 Nontargeted metabolites in PAH
Supplementary Fig. S2 Targeted HPLC measures of metabolites in PAH
Supplementary Fig. S3 Mitochondrial protein expression in PAH
Supplementary Fig. S4 Overall expression of proteins in control PAEC from commercially purchased ($n = 2$) and harvested at Cleveland Clinic ($n = 3$)

Supplementary Methods

Supplementary Table S1. 2,556 proteins quantified from proteomics

Protein	Gene	Fold Change	*P*-value
Aladin	AAAS	0.9074	0.6311
AP2-associated protein kinase 1	AAK1	0.9302	0.3845
Mth938 domain-containing protein	AAMDC	0.9407	0.5520
Angio-associated migratory cell protein	AAMP	0.9774	0.8661
Alanine--tRNA ligase, cytoplasmic	AARS	1.0521	0.6359
BUB3-interacting and GLEBS motif-containing protein ZNF207	ZNF207	1.4153	0.0581
DBIRD complex subunit ZNF326	ZNF326	0.9328	0.7456
Zinc finger Ran-binding domain-containing protein 2	ZRANB2	1.3499	0.0329
Centromere/kinetochore protein zw10 homolog	ZW10	1.3957	0.1836
Zyxin	ZYX	1.2211	0.0653

Fold Change, PAH to healthy controls; *P*-value, PAH *vs*. healthy controls; NA, not available.

Supplementary Table S2. 170 differentially expressed proteins in PAH PAEC compared with healthy control PAEC (all *P* < 0.05)

Gene*	Upregulated (PAH/Controls)	*P*-value	Gene*	Downregulated (PAH/Controls)	*P*-value
UBE2E1	2.5300	0.0331	NPM3	0.2270	0.0436
FOCAD	2.0445	0.0011	**NOS3#**	0.2346	0.0302
EFEMP1	2.0130	0.0196	NXN	0.2841	0.0086
NEXN	1.8559	0.0009	EBP	0.3469	0.0018
RAB27A	1.7973	0.0033	GIT1	0.3785	0.0146
**MTHFD1L#**	1.7884	0.0404	NUP188	0.4698	0.0148
NT5DC1	1.7659	0.0028	POLR2B	0.5073	0.0255
RBM12B	1.7642	0.0002	HGS	0.5296	0.0101
WDR5	1.7529	0.0212	LRPAP1	0.5333	0.0389
MRPS31	1.7364	0.0017	MTCH1	0.5459	0.0145
EIF4G3	1.7114	0.0384	PLCG1	0.5473	0.0249
AASDHPPT	1.6622	0.0201	HDHD1	0.5669	0.0483
PRPF4	1.6554	0.0022	MGST1	0.5780	0.0160
PES1	1.6012	0.0074	NRGN	0.5905	0.0094
MPHOSPH10	1.5886	0.0085	CACNA2D1	0.5961	0.0422
PNO1	1.5878	0.0114	SELT	0.6142	0.0076
**MRPS7**&	1.5765	0.0259	GIMAP1	0.6155	0.0128
GOLPH3	1.5519	0.0496	FKBP2	0.6258	0.0409
ZC3H13	1.5519	0.0407	SEPT6	0.6271	0.0045
ZCCHC8	1.5469	0.0031	SYNE1	0.6274	0.0285
**ZC3H4#**&	1.5377	0.0003	MGARP	0.6358	0.0410
NDUFB7	1.5018	0.0009	PSMG3	0.6379	0.0033
SGTA	1.4872	0.0345	GSN	0.6440	0.0218
USP15	1.4805	0.0131	CD276	0.6477	0.0164
CASP4	1.4773	0.0363	TCIRG1	0.6486	0.0057
SPATS2	1.4752	0.0289	FERMT3	0.6586	0.0010
**EIF2A#**&	1.4691	0.0233	PRKACA	0.6621	0.0294
TGOLN2	1.4642	0.0447	**GNA11#**	0.6665	0.0062
USP10	1.4543	0.0097	RAB8B	0.6776	0.0289
IGF2BP3	1.4536	0.0131	RAB43	0.6784	0.0177
GIPC1	1.4415	0.0221	CSTB	0.6862	0.0094
TM9SF4	1.4288	0.0219	BDH2	0.6889	0.0106
SF3A2	1.4282	0.0083	**PABPN1**	0.6922	0.0448
GIGYF2	1.4262	0.0025	RABIF	0.6964	0.0198
KCTD12	1.4021	0.0317	CTBP2	0.6967	0.0195
**NME1#**	1.3751	0.0261	ACAT2	0.6976	0.0019
NOSIP	1.3714	0.0198	RMDN3	0.6986	0.0484
NACA	1.3695	0.0376	SEP15	0.7009	0.0390
PPIF	1.3687	0.0136	ERGIC2	0.7010	0.0065
USP47	1.3634	0.0342	**SOD1**	0.7019	0.0432
SEC13	1.3631	0.0040	**SNCG#**&	0.7092	0.0496
CDK9	1.3599	0.0240	AP3M1	0.7110	0.0362
ECI1	1.3593	0.0045	NCKAP1	0.7164	0.0069
YARS2	1.3580	0.0310	TMEM189	0.7216	0.0237
**EIF4B#**&	1.3529	0.0187	UROD	0.7229	0.0075
CSRP1	1.3500	0.0459	NRBP1	0.7243	0.0168
ZRANB2	1.3499	0.0329	DPYSL2	0.7270	0.0490
CNN2	1.3382	0.0419	AKT1	0.7279	0.0187
ARMCX3	1.3352	0.0400	TXNDC17	0.7302	0.0312
RPA1	1.3248	0.0171	PMPCA	0.7313	0.0320
PAF1	1.3239	0.0375	CTSL	0.7321	0.0100
**EIF5B**	1.3075	0.0174	UBE2I	0.7324	0.0031
PPID	1.3068	0.0242	GBP1	0.7328	0.0082
NUP160	1.3042	0.0200	CNDP2	0.7343	0.0239
**SHMT2#**	1.3038	0.0366	**STAT1**&	0.7344	0.0095
HMOX2	1.3029	0.0111	TTLL12	0.7389	0.0155
SEPT10	1.2971	0.0377	IDI1	0.7392	0.0199

ADI1	1.2875	0.0410	PLCB3	0.7397	0.0200
FAM96A	1.2851	0.0122	TOLLIP	0.7398	0.0445
RPL3	1.2822	0.0468	C16orf62	0.7420	0.0456
LYAR	1.2793	0.0093	ANO6	0.7434	0.0427
CLPP	1.2677	0.0134	CALM3	0.7488	0.0193
**STOML2**	1.2621	0.0276	APOA1BP	0.7496	0.0094
NUP88	1.2576	0.0371	KIAA0196	0.7548	0.0224
**EIF3C**	1.2571	0.0171	RPL27	0.7571	0.0055
RANGAP1	1.2541	0.0309	RTN3	0.7683	0.0227
WDFY1	1.2533	0.0347	ESYT2	0.7688	0.0180
**HSPA5#**&	1.2485	0.0488	GBE1	0.7706	0.0204
NUP155	1.2456	0.0443	MAPK1	0.7711	0.0109
**HNRNPH1**	1.2447	0.0214	SLC25A1	0.7732	0.0381
MRPL23	1.2408	0.0490	CPPED1	0.7798	0.0164
MRPS28	1.2314	0.0385	TRIP6	0.7846	0.0496
**HSP90B1**	1.2167	0.0492	KRTCAP2	0.7939	0.0227
GRPEL1	1.2124	0.0476	VPS29	0.7945	0.0199
ALDH18A1	1.2121	0.0270	CDIPT	0.7989	0.0379
PSMD1	1.2095	0.0434	KIAA1033	0.7989	0.0150
SDCBP	1.2014	0.0462	VCL	0.7991	0.0293
MESDC2	1.1958	0.0478	LAMC1	0.8019	0.0230
ADSL	1.1923	0.0375	SYNE2	0.8020	0.0355
**XRCC5#**	1.1836	0.0417	VPS13C	0.8052	0.0470
			PFN1	0.8062	0.0350
			EFTUD1	0.8131	0.0238
			RPL36	0.8145	0.0460
			**CAPN1**&	0.8206	0.0327
			GDI1	0.8228	0.0367
			PLIN3	0.8241	0.0347
			DENR	0.8327	0.0446
			GLTP	0.8338	0.0466
			**MOGS#**	0.8356	0.0339
			NUTF2	0.8366	0.0417

*Find names of proteins in Supplementary Table S1; *P*-value, PAH *vs*. healthy controls; Bolded text identifies the proteins selected for targeted proteomics; #, indicates the protein is confirmed in the second cohort of PAH samples (n = 5) by targeted proteomics; &, identifies those that reach significance of $P < 0.05$, one-tailed.

Supplementary Table S3. Canonical pathways of proteins differentially expressed in PAH PAEC

Ingenuity Canonical Pathways	*P*-value of Overlap#	Molecules*
EIF2 signaling	0.0001	RPL27,EIF3C,AKT1,MAPK1,RPL3,EIF5B,EIF4G3,HSPA5,EIF2A
Nitric oxide signaling	0.0004	CALM1 (includes others),HSP90B1,AKT1,MAPK1,PRKACA,NOS3
NOS3 signaling	0.0004	CALM1 (includes others),HSP90B1,NOSIP,AKT1,PRKACA,NOS3,HSPA5
Apelin adipocyte signaling pathway	0.0007	MGST1,MAPK1,GNA11,PRKACA,SOD1
Hypoxia signaling	0.0007	HSP90B1,AKT1,NOS3,UBE2E1,UBE2I
Cholesterol biosynthesis	0.0007	EBP,ACAT2,IDI1
Androgen signaling	0.0011	CALM1 (includes others),MAPK1,GNA11,CACNA2D1,PRKACA,POLR2B
CREB signaling	0.0017	CALM1 (includes others),AKT1,MAPK1,GNA11,CACNA2D1,PRKACA,POLR2B
Protein ubiquitination pathway	0.0020	HSP90B1,USP15,USP47,USP10,PSMD1,HSPA5,UBE2E1,UBE2I
Mevalonate pathway I	0.0022	ACAT2,IDI1
IL-22 signaling	0.0023	AKT1,MAPK1,STAT1
G beta gamma signaling	0.0024	AKT1,MAPK1,GNA11,CACNA2D1,PRKACA
Geranylgeranyldiphosphate biosynthesis I	0.0030	ACAT2,IDI1
Endocannabinoid cancer inhibition pathway	0.0032	AKT1,MAPK1,PRKACA,CASP4,NOS3,EIF2A
Sertoli cell-sertoli cell junction signaling	0.0041	AKT1,MAPK1,PRKACA,VCL,NOS3,RAB8B
Corticotropin releasing hormone signaling	0.0060	CALM1 (includes others),MAPK1,CACNA2D1,PRKACA,NOS3
Inhibition of angiogenesis by TSP1	0.0065	AKT1,MAPK1,NOS3
Apelin endothelial signaling pathway	0.0066	CALM1 (includes others),AKT1,MAPK1,GNA11,NOS3
Adrenomedullin signaling pathway	0.0074	CALM1 (includes others),AKT1,MAPK1,GNA11,PRKACA,NOS3
Citrulline metabolism	0.0078	ALDH18A1,NOS3
Glucocorticoid receptor signaling	0.0081	HSP90B1,AKT1,MAPK1,PRKACA,POLR2B,HSPA5,STAT1,UBE2I
Sirtuin signaling pathway	0.0100	PPID,AKT1,MAPK1,NDUFB7,SOD1,NOS3,XRCC5
Regulation of eIF4 and p70S6K signaling	0.0102	EIF3C,AKT1,MAPK1,EIF4G3,EIF2A
Relaxin signaling	0.0107	AKT1,MAPK1,GNA11,PRKACA,NOS3
Sumoylation pathway	0.0112	GDI1,CTBP2,RPA1,UBE2I
RANK signaling	0.0112	CALM1 (includes others),AKT1,MAPK1,GSN
IL-1 signaling	0.0112	TOLLIP,MAPK1,GNA11,PRKACA
Methylthiopropionate biosynthesis	0.0123	ADI1
NOS2 signaling	0.0129	CALM1 (includes others),MAPK1,STAT1
GNRH signaling	0.0129	CALM1 (includes others),MAPK1,GNA11,CACNA2D1,PRKACA
Amyloid processing	0.0138	AKT1,MAPK1,PRKACA
Opioid signaling pathway	0.0141	CALM1 (includes others),AKT1,MAPK1,CACNA2D1,PRKACA,NOS3
Apelin cardiomyocyte signaling pathway	0.0145	AKT1,MAPK1,GNA11,NOS3
VEGF signaling	0.0155	AKT1,MAPK1,VCL,NOS3
Germ cell-sertoli cell junction signaling	0.0174	AKT1,MAPK1,VCL,GSN,RAB8B
Apelin muscle signaling pathway	0.0182	AKT1,NOS3
Unfolded protein response	0.0191	HSP90B1,HSPA5,EIF2A

#The overlap *P*-value measures whether there is a statistically significant overlap between the dataset genes and the genes that are regulated by a transcriptional regulator. It is calculated using Fisher's Exact Test, and significance is generally attributed to *P*-values < 0.01; *Find names of proteins in Supplementary Table S1.

Supplementary Table S4. Causal networks of proteins differentially expressed in PAH PAEC

Master Regulator	Molecule Type	*P*-value of Overlap	Target Molecules in Dataset*
CDC73	other	0.0015	GBP1, STAT1
mir-223	microRNA	0.0023	AKT1, GBP1, GLTP, HNRNPH1, HSP90B1, HSPA5
C1QBP	transcription regulator	0.0024	AKT1, GBP1, HNRNPH1, HSP90B1, HSPA5, SNCG, STAT1
EPAS1	transcription regulator	0.0027	AKT1, GBP1, HNRNPH1, HSP90B1, HSPA5, NOS3, SNCG
EGFR	kinase	0.0031	AKT1, GBP1, HNRNPH1, HSP90B1, HSPA5
WT1	transcription regulator	0.0099	GSN, HSP90B1, MGST1, SEC13
mir-374	microRNA	0.0124	AKT1
SRI	transporter	0.0124	HSPA5
SLC20A1	transporter	0.0124	AKT1
CCNH	transcription regulator	0.0124	CTBP2
MYO1C	enzyme	0.0124	GSN

*Find names of proteins in Supplementary Table S1.

Supplementary Table S5. Biological functions of proteins differentially expressed in PAH PAEC

Categories	Diseases or Functions Annotation	*P*-value of Overlap	Molecules*
Cellular Compromise,Inflammatory Response	Degranulation of cells	<0.0001	CALM1 (includes others),CNN2,CSTB,FERMT3,GSN,HMOX2,MAPK1,MGST1,PSMD1,RAB27A,SDCBP,SOD1,TCIRG1,TOLLIP,VCL,VPS35L,XRCC5
Cellular Compromise,Inflammatory Response	Degranulation of neutrophils	0.0001	CNN2,CSTB,GSN,HMOX2,MAPK1,MGST1,PSMD1,RAB27A,SDCBP,TCIRG1,TOLLIP,VCL,VPS35L,XRCC5
Protein Synthesis	Metabolism of protein	0.0001	AKT1,CASP4,CSTB,CTSL,EIF2A,EIF3C,EIF4B,EIF4G3,EIF5B,GOLPH3,GSN,HGS,HSP90B1,LAMC1,MRPL23,PFN1,RPL27,RPL3,SOD1,UBE2E1,UBE2I
Protein Synthesis	Synthesis of protein	0.0004	EIF2A,EIF3C,EIF4B,EIF4G3,EIF5B,GOLPH3,MRPL23,RPL27,RPL3,SOD1
Immunological Disease	Hypersensitive reaction	0.0012	AKT1,CASP4,CTSL,GSN,IDI1,NOS3,STAT1,SYNE2,UBE2I,VCL
Cell Death and Survival	Cell death of cervical cancer cell lines	0.0022	AKT1,CASP4,CDK9,GNA11,HNRNPH1,MAPK1,MTCH1,RPA1,SOD1,STAT1,TOLLIP,UBE2I
Infectious Diseases	Infection of cells	0.0030	AKT1,GOLPH3,HGS,HNRNPH1,HSP90B1,HSPA5,MRPL23,NCKAP1,NDUFB7,NRBP1,NUP160,PAF1,RAB8B,RPL3,RTN3,STAT1,UBE2E1
RNA Post-Transcriptional Modification	Processing of RNA	0.0031	HNRNPH1,LYAR,MPHOSPH10,PABPN1,PAF1,PES1,POLR2B,PRKACA,PRPF4,RPL27
Cell Death and Survival	Cell death of tumor cell lines	0.0033	ADI1,AKT1,CASP4,CDK9,CNN2,CTBP2,EFEMP1,EIF2A,EIF4B,GNA11,GSN,HNRNPH1,HSPA5,MAPK1,MTCH1,NOS3,PAF1,PPID,PRKACA,RPA1,SOD1,STAT1,STOML2,SYNE1,TOLLIP,TRIP6,UBE2I,USP10,USP47,WDR5,XRCC5
Cell Death and Survival	Apoptosis of cervical cancer cell lines	0.0036	AKT1,CASP4,CDK9,GNA11,HNRNPH1,MAPK1,MTCH1,RPA1,STAT1,UBE2I
Dermatological Diseases and Conditions,Inflammatory Disease,Inflammatory Response,Organismal Injury and Abnormalities	Dermatitis	0.0037	AKT1,CTSL,GSN,IDI1,NOS3,PSMD1,STAT1,SYNE2,UBE2I,VCL
Infectious Diseases	Viral infection	0.0037	AKT1,CACNA2D1,CTBP2,CTSL,EIF3C,FERMT3,GBP1,GOLPH3,HGS,HNRNPH1,HSP90B1,HSPA5,LRPAP1,MAPK1,MRPL23,NCKAP1,NDUFB7,NRBP1,NUP160,PAF1,PRKACA,RAB8B,RPL3,RTN3,STAT1,TCIRG1,UBE2E1,UBE2I
Infectious Diseases	Infection of tumor cell lines	0.0045	AKT1,GOLPH3,HGS,HSP90B1,MRPL23,NDUFB7,NRBP1,NUP160,PAF1,RAB8B,STAT1,UBE2E1
Cell Death and Survival	Apoptosis of tumor cell lines	0.0046	ADI1,AKT1,CASP4,CDK9,CNN2,CTBP2,EFEMP1,EIF2A,EIF4B,GNA11,GSN,HNRNPH1,HSPA5,MAPK1,MTCH1,NOS3,PPID,PRKACA,RPA1,SOD1,STAT1,STOML2,UBE2I,USP10,WDR5,XRCC5
Hereditary Disorder,Organismal Injury and Abnormalities,Skeletal and Muscular Disorders	Hereditary myopathy	0.0068	ALDH18A1,CACNA2D1,GOLPH3,GSN,HSP90B1,HSPA5,PABPN1,SDCBP,SYNE1,SYNE2,VCL,WASHC5
Cell Death and Survival	Apoptosis	0.0072	ADI1,AKT1,CASP4,CDK9,CNN2,CTBP2,EFEMP1,EIF2A,EIF4B,GNA11,GOLPH3,GSN,HMOX2,HNRNPH1,HSP90B1,HSPA5,LRPAP1,MAPK1,MTCH1,NCKAP1,NOS3,PPID,PRKACA,RPA1,SNCG,SOD1,STAT1,STOML2,UBE2I,USP10,USP47,WDR5,XRCC5

Infectious Diseases	Infection by RNA virus	0.0076	AKT1,CACNA2D1,GOLPH3,HGS,HNRNPH1,HSP90B1,HSPA5,MRPL23,NCKAP1,NDUFB7,NRBP1,NUP160,PAF1,RAB8B,RPL3,RTN3,STAT1,UBE2E1
Cellular Movement	Cell movement of breast cancer cell lines	0.0079	AKT1,CTSL,GIPC1,GNA11,GOLPH3,LRPAP1,MAPK1,PFN1,SDCBP,UBE2I
Neurological Disease	Progressive neurological disorder	0.0139	CALM1 (includes others),CSTB,GOLPH3,GSN,HMOX2,HSP90B1,HSPA5,MAPK1,NAXE,PFN1,RPL3,SNCG,SOD1
Cell Death and Survival	Cell death of breast cancer cell lines	0.0140	AKT1,EIF4B,HSPA5,MAPK1,NOS3,PPID,PRKACA,SOD1,STAT1,USP47
Cellular Movement	Cell movement of tumor cell lines	0.0163	AKT1,CNDP2,CTBP2,CTSL,EFEMP1,FERMT3,GDI1,GIPC1,GNA11,GOLPH3,HSP90B1,LRPAP1,MAPK1,PFN1,PPID,SDCBP,SNCG,STAT1,TRIP6,UBE2I
Cell Death and Survival	Necrosis	0.0172	ADI1,AKT1,CASP4,CDK9,CNN2,CTBP2,EFEMP1,EIF2A,EIF4B,GNA11,GSN,HMOX2,HNRNPH1,HSPA5,LRPAP1,MAPK1,MTCH1,NOS3,PAF1,PPID,PRKACA,RPA1,SOD1,STAT1,STOML2,SYNE1,TOLLIP,TRIP6,UBE2I,USP10,USP47,WDR5,XRCC5

*Find names of proteins in Supplementary Table S1.

Supplementary Table S6. Top diseases and functions in networks of proteins differentially expressed in PAH PAEC

Networks	Top Diseases and Functions	Focus Molecules#	Molecules* in Network
1	Infectious diseases, hematological system development and function, cardiovascular system development and function	16	**ADSL**, **AKT1**, APBB3, APP, **CALM1** (includes others), CCL5, CDC73, **CDK9**, FGF7, GATA2, **GBP1**, **GNA11**, GRN, HBA1/HBA2, HLA-E, **HMOX2**, HPRT1, IDE, IFIT3, IFITM3, **LRPAP1**, LY96, **MAPK1**, **NOS3**, NRP2, OASL, **PAF1**, **PRKACA**, PROC, **RTN3**, SGPL1, SLC4A7, **STAT1**, **SYNE2**, **UBE2I**
2	Cellular compromise, cellular function and maintenance, cancer	15	BAP1, **CASP4**, CCND1, **CSTB**, CXXC1, E2F4, **ECI1**, **EIF2A**, H2AFZ, **HSP90B1**, **HSPA5**, IL7, KMT2C, MEN1, NPAT, PA2G4, **PABPN1**, PALLD, **PFN1**, **POLR2B**, **RAB8B**, **RABIF**, **RMDN3**, **RPL27**, RPL27A, RPL30, SLC37A4, SPEN, SRI, TADA3, TP53,TPI1, TUBB, **WDR5**, **ZC3H13**
3	Cellular movement, cell-to-cell signaling and interaction, hematological system development and function	14	CCNH, CD2, CD44, **CNDP2**, **CTBP2**, CTSS, **EFEMP1**, **EIF4B**, ESR1 F3, **FERMT3**, **GOLPH3**, IGFBP2, **LAMC1**, LMO3, LY96, MAPK14, MITF, **NCKAP1**, NFAT5, **NPM3**, PIK3CG, **PRPF4**, PRPF8, RAC1, RIPK2, SCARB1, TNF, **TOLLIP**, TP63, **TRIP6**, **VCL**, WASF2, **XRCC5**, YBX1
4	Cell cycle, organismal injury and abnormalities, tissue morphology	14	AR, **ARMCX3**, CCDC22, CCDC93, CSF2, **CSRP1**, ESR2, EZH2, **GSN**, HNRNPA2B1, **KCTD12**, MAPK13, MYO1C, **NACA**, NR3C1, **NRBP1**, OGA, **PPID**, **PSMD1**, PTGES3, **RAB27A**, **RAB43**, RBM3, SCARB1, SNX1, SORL1, SPHK1, TGFB1, VPS26A, **VPS29**, VPS35, **VPS35L**, WASHC2A/WASHC2C, **WASHC4**, **WASHC5**
5	Cell death and survival, cell-to-cell signaling and interaction, cellular development	13	BAG3, BAX, BCL2, CAV1, **CD276**, **CTSL**, EBI3, **ESYT2**, ESYT3, FGFR1, GLI1, HES1, IFNG, IGF2R, KRAS, KRT19, LGALS3, LGALS3BP, **MGST1**, **MTCH1**, **NDUFB7**, P2RX7, PSEN1, **RPA1**, **RPL3**, **SDCBP**, SLC29A1, SMARCA2, SOCS1, **SOD1**, SPN, **STOML2**, **SYNE1**, **TCIRG1**, TNFRSF10B
6	Cellular movement, cancer, organismal injury and abnormalities	13	ACTN4, **AKT1**, APPL1, AR, ARFGEF1, ARFGEF2, BUB1B, CDH2, **CNN2**, CTNNB1, DNAJB6, **EBP**, **EIF4G3**, EPAS1, ERG, ESR2, **FKBP2**, **GDI1**, **GIPC1**, **GLTP**, HDAC3, **HGS**, IGF1R, IL2RB,JUND, LITAF, **NOS3**, PELP1, PRKAA1, **SEC13**, **SNCG**, SP1, SUZ12, **USP10**, VPS35
7	Cancer, cell cycle, cellular development	12	**ALDH18A1**, APC, ARAF, CAV1, **CDIPT**, **CIAO2A**, DDX5, DHX9, E2F6, **EIF5B**, FBL, HK2, HMGA1, **HNRNPH1**, HNRNPU, HRAS, **IDI1**, IGFBP2, KDM5B, KIT, KMT2A, let-7, **MOGS**, MYC, NRG1, **NUTF2**, **PES1**, **PNO1**, PROM1, SSB, STUB1, TERT, **UBE2I**, **USP47**, YY1

#Differentially expressed proteins; *Find names of proteins in bold in Supplementary Table S1.

Supplementary Table S7. 3,609 peptides quantified from phosphoproteomics

Protein	Gene	Peptide Sequence	#	Positions[&]	Fold Change	*P*-value
AP2-associated protein kinase 1	AAK1	VQTTPPPAVQGQK	1	606	0.9094	0.5095
AP2-associated protein kinase 1	AAK1	VGSITPPSSPK	2	620	1.0606	0.7773
AP2-associated protein kinase 1	AAK1	VGSITPPSSPK	2	623	1.0606	0.7773
AP2-associated protein kinase 1	AAK1	IISDVTHSAVFGVPASK	1	637	0.9866	0.9474
AP2-associated protein kinase 1	AAK1	RIISDVTHSAVFGVPASK	1	640	1.4153	0.2570
Zyxin	ZYX	VSSIDIEIDSISSIIDDMTK	1	150	2.1871	0.2744
Zyxin	ZYX	GPPASSPAPAPK	1;2	259	0.8691	0.6629
Zyxin	ZYX	FSPVTPK	1;2	267	1.3213	0.2977
Zyxin	ZYX	FSPGAPGGSGSQPNQK	1	281	0.9317	0.6388
Zyxin	ZYX	SPGAPGPITIK	1	344	0.8679	0.4847

#, number of phosphorylation sites on that specific peptide; [&]The amino acid residue within the protein that is phosphorylated; Fold Change, PAH to healthy controls; *P*-value, PAH *vs*. healthy controls; NA, not available.

Supplementary Table S8. 240 differentially expressed phosphopeptides in PAH PAEC compared with healthy control PAEC (all *P* < 0.05)

Gene*	Positions[&]	Up regulated (PAH/ Controls)	*P*-value	Proteome (Fold Change)	Gene*	Positions [&]	Down regulated (PAH/ Controls)	*P*-value	Proteome (Fold Change)
SOGA1	141	5.2073	0.0163		MTOR	1166	0.1981	0.0019	
SOGA1	142	5.2073	0.0163		TMX1	247	0.1988	0.0108	0.8854
HNRNPA1	4	4.7206	0.0006	0.9915	AFAP1	668	0.2635	0.0487	
PNN	381	4.4304	0.0076	1.074	TNKS1BP1	429	0.2887	0.0072	1.2301
PKN1	69	4.364	0.0131	0.6701	NAV1	257	0.2916	0.0042	
MYL9;MYL12A;MYL12B	20	4.2779	0.0001		EIF5B	107	0.3214	0.0011	1.3075
MYL9;MYL12A;MYL12B	19	4.2779	0.0001		TNIK	594	0.3347	0.0169	1.1426
DKC1	455	4.1997	0.0038	1.1699	AHNAK	5720	0.3481	0.0017	1.0128
IWS1	196	4.0305	0.0291		SEC22B	137	0.3529	0.0058	1.1563
IWS1	198	4.0305	0.0291		FAM129B	679	0.3681	0.0196	1.018
NES	471	3.9805	0.0259	1.2992	GATAD2B	135	0.3936	0.0005	
ANAPC1	547	3.4354	0.0137		BRD8	128	0.4187	0.0257	
ANAPC1	563	3.4354	0.0137		TBC1D2	460	0.4285	0.0026	
PHLDB2	493	3.4174	0.0222		COPB2	830	0.4299	0.0113	1.0041
PPFIBP1	280	3.2502	0.0023		ITPR3	1832	0.4299	0.01	0.8944
SACS	3510	3.163	0.0184		PDLIM7	111	0.431	0.0002	1.1093
FAM117A	213	3.0591	0.0433		RPLP2	79	0.4312	0.0247	0.9449
SEPT2	218	2.998	0.0002	1.0307	ANXA2;ANXA2P2	22	0.4409	0.0308	
ZBTB21	778	2.994	0.0228		MAP1B	831	0.4444	0.0035	0.9811
BAG3	289	2.8356	0.0099	0.9911	MAP1B	832	0.4444	0.0035	0.9811
CHCHD3	50	2.7993	0.0072	0.9942	SPECC1L	384	0.4483	0.0265	0.9528
MYCT1	138	2.7727	0.0028	1.045	HDGF	125	0.4518	0.0207	1.1123
GORASP2	383	2.7657	0.0005	1.1631	ANXA1	5	0.4542	0.0057	0.9903
C11orf96	399	2.536	0.0343		CAAP1	167	0.4546	0.0471	
MAP3K11	451	2.4548	0.0032		DCP1A	277	0.4626	0.0401	
ADD3	647	2.4202	0.0387	0.7326	DCP1A	281	0.4626	0.0401	
SASH1	813	2.4095	0.0188	1.1726	CDK16	153	0.4701	0.0235	
ARHGEF6	530	2.3761	0.0264		PLCL1	556	0.4709	0.0034	
OSBPL3	410	2.3465	0.0388	1.6187	NIPBL	2658	0.4717	0.0408	
GNG12	26	2.2892	0.0415	0.9847	BIN1	272	0.4728	0.0393	
MYCT1	135	2.2805	0.0458	1.045	MTA1	559	0.4735	0.0217	
HSPA12B	46	2.2016	0.0398		ACACA	23	0.4764	0.0014	0.9836
PLEC	4396;4282;4259	2.1967	0.0313	1.1045	HDGF	126	0.4803	0.0246	1.1123
ZNF609	1055	2.1818	0.026		HNRNPA1	2	0.4827	0.0335	0.9915
VIM	63	2.1504	0.0147	0.9248	JUP	665	0.4864	0.0034	1.2949
TBC1D9B	435	2.1215	0.0357	0.9332	MAP1B	1801	0.4965	0.0028	0.9811
SDPR	43	2.1185	0.0026	0.8585	UBE2O	515	0.4991	0.0044	
WDR44	96	2.1071	0.0267		RPS6KA4	675	0.5029	0.0048	
P4HA3	18	2.0869	0.0228		TMSB4X	2	0.5156	0.0206	0.711
TOR1AIP1	143	2.0843	0.0084		MYH10	1952	0.5205	0.0047	0.8855
RRM2	20	2.0782	0.0418		NFATC2	107	0.5341	0.0025	
NES	831	2.0743	0.0239	1.2992	SYNJ1	1057	0.5353	0.023	
CTNNA1;CTNNA2	285	2.0205	0.0421		DBNL	167	0.5355	0.0175	
JUN	62	2.011	0.0254		ELMSAN1	698	0.5366	0.005	
FOXK1	60	1.9988	0.0345		PGM3	64	0.5366	0.0321	0.8828
MAP4	507	1.9956	0.0124	1.0107	SYNPO	812	0.5379	0.0035	0.9516

LPXN	19	1.9931	0.0013	1.1444	IGF2BP2	99	0.5382	0.0279	1.2311
SRRM2	1112	1.9755	0.0216	1.205	SART1	474	0.5421	0.0362	1.098
TJP2	151	1.9584	0.0071		SART1	486	0.5421	0.0362	1.098
HNRNPD	82	1.9532	0.0007	1.154	TXLNA	515	0.5462	0.0143	0.9496
ACVRL1	160	1.9451	0.0343		RPS6KA4	676	0.5466	0.0307	
ERCC5	562	1.927	0.0395		PAXBP1	262	0.5539	0.0474	
ERCC5	563	1.927	0.0395		NIFK	223	0.5556	0.0421	1.277
ROCK2	1137	1.9216	0.0472	1.0363	TBC1D10B	658	0.56	0.0081	
RPRD2	641	1.9066	0.0439		SDPR	37	0.5609	0.0252	0.8585
VAMP4	30	1.903	0.0187		ARHGEF2	147	0.5656	0.0276	
MACF1	5511	1.8977	0.0324		TGOLN2	296	0.5661	0.0081	**1.4642**
FAM21C	484	1.8899	0.0127		FLNC	2200	0.5679	0.0104	0.9512
RAB11FIP5	359	1.8613	0.0262		ACVRL1	161	0.5702	0.0432	
TJP2	147	1.861	0.0254		CEP170	783	0.5722	0.0496	
CDK17	122	1.8474	0.0119		PARP4	1504	0.5734	0.0453	0.8527
TNRC18	1857	1.8346	0.0079		SMTN	792	0.5749	0.0055	
FAM134A	330	1.8087	0.0441		SHANK3	254	0.5831	0.0308	0.8023
OSBPL11	172	1.7964	0.0337		PUM1	683	0.5847	0.0435	1.5826
ATG9A	14	1.793	0.0378		ATXN2L	111	0.5866	0.0261	1.4078
DCAF6	657	1.7754	0.0122		FKBP15	1154	0.587	0.0186	1.1182
PPP2R5E	6	1.772	0.0485		HN1L	81	0.5879	0.046	1.1219
PAFAH1B2	2	1.7618	0.0365	0.8308	ITSN1	315	0.6069	0.0217	
SMN1	28	1.7549	0.0216		FGD5	552	0.6106	0.0063	
GYS1	663	1.7394	0.0474		AP3D1	489	0.6115	0.0345	0.961
PLEKHG5	903	1.7179	0.0248		ZNF185	466	0.6123	0.017	1.0074
NUFIP2	629	1.7175	0.0235		SNX30	40	0.6135	0.0101	
GORASP2	347	1.7139	0.0375	1.1631	ATG16L1	143	0.6141	0.0047	
CTNNA1	264	1.7124	0.0251	1.0082	KHDRBS1	20	0.6147	0.0458	1.3572
SNTB2	233	1.711	0.0441	0.687	CEP170	860	0.6153	0.0205	
PTPN14	578	1.6994	0.0207	1.4866	ERRFI1	251	0.6209	0.0286	
CDH6	786	1.6991	0.0427		RRAGC;RRAGD	95	0.6226	0.0304	
CTTNBP2NL	568	1.6985	0.0085		LIMD1	272	0.6231	0.0474	
AHNAK	3426	1.6936	0.022	1.0128	MAST4	1176	0.6239	0.037	
RANBP3	265	1.6741	0.0268		EIF5B	113	0.6287	0.0337	**1.3075**
SDPR	364	1.66	0.0115	0.8585	NFATC2	106	0.6318	0.0405	
SACS	3514	1.6578	0.0172		LARP7	261	0.632	0.037	
DDX55	201	1.6497	0.011		GPSM1	470	0.6339	0.0287	
NES	1521	1.646	0.0162	1.2992	SDPR	284	0.634	0.0052	0.8585
FLNB	2298	1.6455	0.0089	1.1969	NCK1	21	0.6365	0.0338	
AHNAK	511	1.6422	0.0257	1.0128	MTMR12	454	0.637	0.0178	
EXOC7	250	1.6422	0.0057	0.5979	RIN2	366	0.6378	0.0103	
RAB12	21	1.642	0.0311		PCNP	147	0.6399	0.0241	1.3716
PRRC2A	808	1.6336	0.0368	1.3972	DOCK5	1789	0.64	0.0206	
PTRF	365	1.631	0.0222	1.2518	RIOK2	335	0.6463	0.0432	
MAP4	521	1.6236	0.0299	1.0107	MAP4K4	872	0.6471	0.042	
PRKD3	41	1.6184	0.0462		CEP131	96	0.653	0.0277	
TRIM28	518	1.6161	0.048	1.0914	FKBP15	1152	0.6531	0.0097	1.1182
CHMP3	134	1.6032	0.0159		MAP7D1	118	0.6547	0.0339	
CCDC86	21	1.6022	0.0348		SIRT1	16	0.6633	0.0453	
DNAJC5	10	1.5948	0.0232		SSFA2	668	0.6642	0.0471	
UBAP2L	598	1.5793	0.0268	1.3112	AHNAK	41	0.6654	0.04	1.0128
SCAF11	293	1.5634	0.0188		SRRM2	875	0.667	0.0416	1.205
APPL1	401	1.5537	0.0339	0.9269	MIB2	309	0.6678	0.0152	
SCRIB	772	1.5464	0.0178		USH2A	2552	0.6717	0.0453	
PA2G4	2	1.546	0.0118	1.0456	TOP2A	1247	0.6725	0.0182	
NSFL1C	114	1.5441	0.0113	1.2115	PHRF1	1202	0.6769	0.0326	
ZC3H4	1114	1.5372	0.037	**1.5377**	LARP1	75	0.6775	0.0164	0.8324
TRIM56	469	1.5311	0.0275		G3BP2	225	0.6864	0.0282	1.0487
FLII	801	1.5172	0.0242	1.0318	AHNAK	570	0.688	0.0267	1.0128
PXN	130	1.5165	0.0347	0.9571	PFDN4	125	0.6972	0.0263	1.111

USP8	612	1.5154	0.0193		PLEKHA1	308	0.6979	0.0247	
PABPN1	177	1.5112	0.0215	**0.6922**	PIKFYVE	210	0.6984	0.0141	
HDAC7	67	1.5107	0.0364		NAA10	194	0.699	0.0164	1.0659
DDHD1	305	1.4968	0.0362		TP53BP1	830	0.7059	0.0207	1.1084
FOXK1	265	1.4913	0.0337		ATP2B1	1119	0.7173	0.0401	
SRRM2	866	1.4812	0.0228	1.205	HNRNPA0	188	0.7214	0.0469	1.1023
FAM114A1	196	1.48	0.0167	1.1058					
CTNNA1;CTNNA2	284	1.4763	0.0299						
SCRIB	1367	1.4737	0.0119						
RABL6	470	1.4666	0.0457						
RABL6	471	1.4666	0.0457						
FAM122A	270	1.466	0.0136						
CCAR2	484	1.4606	0.0406	0.9944					
GTPBP1	24	1.453	0.0408						
LRRFIP1	83	1.4354	0.0273	1.1816					
XPO6	190	1.4312	0.046						
CCDC43	139	1.4302	0.0449						
PDS5B	1370	1.4252	0.0285	1.0796					
SRRM2	2121	1.4225	0.0472	1.205					
PCM1	93	1.3833	0.0476						
EEF2K	18	1.3505	0.0371						
ATRX	473	1.3321	0.0499						

*Find names of proteins and peptide sequence in Supplementary Table S7; [&]The amino acid residue within the protein that is phosphorylated; *P*-value, PAH *vs*. healthy controls; Proteome (fold change), ratio of PAH to healthy controls, in bold if *P*-value less than 0.05 PAH *vs*. healthy controls.

Supplementary Table S9. Canonical pathways of phosphoproteins differentially expressed in PAH PAEC

**Ingenuity Canonical Pathways**	***P*-value of Overlap**	**Molecules***
Signaling by Rho family GTPases	0.0004	MYL9,ROCK2,JUN,ARHGEF6,PIKFYVE,VIM,ARHGEF2,PKN1,GNG12,SEPT2
ILK signaling	0.0019	MYL9,FLNB,MTOR,PXN,JUN,ARHGEF6,VIM,TMSB10/TMSB4X
RhoA signaling	0.0030	MYL9,ROCK2,PLEKHG5,PIKFYVE,PKN1,SEPT2
CXCR4 signaling	0.0032	MYL9,ROCK2,PXN,JUN,ITPR3,PRKD3,GNG12
Phospholipase C signaling	0.0040	MYL9,AHNAK,ITPR3,ARHGEF6,HDAC7,ARHGEF2,PRKD3,GNG12
Thrombin signaling	0.0100	MYL9,ROCK2,ITPR3,ARHGEF6,ARHGEF2,PRKD3,GNG12
Cholecystokinin/Gastrin-mediated signaling	0.0112	ROCK2,PXN,JUN,ITPR3,PRKD3
Caveolar-mediated Endocytosis signaling	0.0112	FLNB,ITSN1,COPB2,CAVIN1
RhoGDI signaling	0.0120	MYL9,ROCK2,ARHGEF6,PIKFYVE,ARHGEF2,GNG12
Ephrin B signaling	0.0120	ROCK2,PXN,ITSN1,GNG12
IL-8 signaling	0.0141	MYL9,ROCK2,MTOR,JUN,PRKD3,MAP4K4,GNG12

*Find names of proteins in Supplementary Table S7.

Supplementary Table S10. Causal networks of phosphoproteins differentially expressed in PAH PAEC

Master Regulator	Molecule Type	*P*-value of Overlap	Target Molecules in Dataset*
ESR2	ligand-dependent nuclear receptor	0.0067	JUN,MTOR
DYRK1A	kinase	0.0344	SIRT1
DYRK3	kinase	0.0344	SIRT1
PPP2R2D	phosphatase	0.0344	ACACA
FLNA	other	0.0344	VIM
PRKCQ	kinase	0.0344	ARHGEF6
NEU3	enzyme	0.0422	FAM129B,JUN,PXN

*Find names of proteins in Supplementary Table S7.

Supplementary Table S11. Biological functions of phosphoproteins differentially expressed in PAH PAEC

Categories	Diseases or Functions Annotation	*P*-value of Overlap	Molecules*
Cancer,organismal injury and abnormalities	Incidence of tumor	0.0003	ACVRL1,ADD3,AFAP1,AHNAK,ANXA1,ANXA2,AP3D1,APPL1,ARHGEF2,ARHGEF6,ATG16L1,ATG9A,ATP2B1,ATRX,ATXN2L,BIN1,BRD8,CAVIN1,CAVIN2,CCAR2,CDK16,CEP170,CHCHD3,COPB2,CTNNA1,CTTNBP2NL,DBNL,DDHD1,EEF2K,EIF5B,ERCC5,ERRFI1,FGD5,FKBP15,FLII,FLNB,G3BP2,GNG12,GORASP2,HDAC7,HDGF,HSPA12B,ITPR3,ITSN1,JUN,JUP,KHDRBS1,LARP1,LARP7,LIMD1,LPXN,LRRFIP1,MACF1,MAP4,MAP4K4,MAST4,MTA1,MTMR12,MTOR,MYCT1,MYL9,NAV1,NES,NIPBL,OSBPL3,PA2G4,PARP4,PCM1,PDLIM7,PHLDB2,PHRF1,PIKFYVE,PKN1,PLEC,PLEKHG5,PNN,PPFIBP1,PRRC2A,PUM1,PXN,RAB11FIP5,RAB12,RABL6,RANBP3,RIN2,RIOK2,ROCK2,RPLP2,RPRD2,RRM2,SART1,SASH1,SCAF11,SCRIB,SEC22B,SHANK3,SMTN,SPECC1L,SYNPO,TBC1D9B,TJP2,TMSB10/TMSB4X,TNIK,TNKS1BP1,TOP2A,TRIM28,TXLNA,USH2A,USP8,VIM,WASHC2A/WASHC2C,WDR44,XPO6
Cancer,organismal injury and abnormalities	Head and neck carcinoma	0.0004	ACVRL1,ADD3,AFAP1,AHNAK,ANXA1,ANXA2,AP3D1,APPL1,ARHGEF2,ARHGEF6,ATG16L1,ATG9A,ATP2B1,ATRX,ATXN2L,BIN1,BRD8,CAAP1,CCAR2,CCDC86,CDK16,CEP170,COPB2,CTNNA1,CTTNBP2NL,DBNL,DDHD1,DDX55,EEF2K,EIF5B,ERCC5,FAM114A1,FAM117A,FAM129B,FGD5,FKBP15,FLII,FLNB,FOXK1,G3BP2,GATAD2B,GORASP2,HDAC7,HDGF,HSPA12B,ITPR3,ITSN1,JUN,JUP,KHDRBS1,LARP1,LARP7,LIMD1,LPXN,LRRFIP1,MACF1,MAP4,MAP4K4,MAST4,MIB2,MTA1,MTMR12,MTOR,MYCT1,MYL9,NAA10,NAV1,NES,NIPBL,NUFIP2,OSBPL11,P4HA3,PA2G4,PABPN1,PAFAH1B2,PARP4,PCM1,PDLIM7,PFDN4,PHLDB2,PHRF1,PIKFYVE,PKN1,PLEC,PLEKHG5,PNN,PPFIBP1,PRKD3,PRRC2A,PUM1,PXN,RAB11FIP5,RABL6,RANBP3,RETREG2,RIN2,RIOK2,ROCK2,RPLP2,RPRD2,RRAGC,RRM2,SART1,SASH1,SCAF11,SCRIB,SEPT2,SHANK3,SIRT1,SMN1/SMN2,SMTN,SNTB2,SPECC1L,SYNPO,TBC1D9B,TJP2,TMSB10/TMSB4X,TMX1,TNIK,TNKS1BP1,TOP2A,TRIM28,TRIM56,TXLNA,UBE2O,USH2A,USP8,VIM,WASHC2A/WASHC2C,WDR44,XPO6
Cancer,organismal injury and abnormalities	Neck neoplasm	0.0005	ACVRL1,ADD3,AFAP1,AHNAK,ANXA1,ANXA2,AP3D1,APPL1,ARHGEF2,ARHGEF6,ATG16L1,ATG9A,ATP2B1,ATRX,ATXN2L,BIN1,BRD8,CAAP1,CCAR2,CDK16,CEP170,COPB2,CTNNA1,CTTNBP2NL,DBNL,DDHD1,DDX55,EEF2K,EIF5B,ERCC5,FAM114A1,FAM117A,FAM129B,FGD5,FKBP15,FLII,FLNB,FOXK1,G3BP2,GATAD2B,GORASP2,HDAC7,HDGF,HSPA12B,ITPR3,ITSN1,JUP,KHDRBS1,LARP1,LARP7,LIMD1,LPXN,LRRFIP1,MACF1,MAP4,MAP4K4,MAST4,MIB2,MTA1,MTMR12,MTOR,MYCT1,MYL9,NAA10,NAV1,NES,NIPBL,OSBPL11,P4HA3,PA2G4,PABPN1,PAFAH1B2,PARP4,PCM1,PDLIM7,PFDN4,PHLDB2,PHRF1,PIKFYVE,PKN1,PLEC,PLEKHG5,PNN,PPFIBP1,PRKD3,PRRC2A,PUM1,PXN,RAB11FIP5,RABL6,RANBP3,RETREG2,RIN2,RIOK2,ROCK2,RPLP2,RPRD2,RRAGC,RRM2,SART1,SASH1,SCAF11,SCRIB,SEPT2,SHANK3,SIRT1,SMN1/SMN2,SMTN,SNTB2,SPECC1L,SYNPO,TBC1D9B,TJP2,TMSB10/TMSB4X,TMX1,TNIK,TNKS1BP1,TOP2A,TRIM28,TRIM56,TXLNA,UBE2O,USH2A,USP8,VIM,WASHC2A/WASHC2C,WDR44,XPO6
cCancer,organismal injury and abnormalities	Head and neck tumor	0.0005	ACVRL1,ADD3,AFAP1,AHNAK,ANXA1,ANXA2,AP3D1,APPL1,ARHGEF2,ARHGEF6,ATG16L1,ATG9A,ATP2B1,ATRX,ATXN2L,BAG3,BIN1,BRD8,CAAP1,CCAR2,CCDC86,CDK16,CEP170,COPB2,CTNNA1,CTTNBP2NL,DBNL,DDHD1,DDX55,EEF2K,EIF5B,ERCC5,FAM114A1,FAM117A,FAM129B,FGD5,FKBP15,FLII,FLNB,FOXK1,G3BP2,GATAD2B,GORASP2,HDAC7,HDGF,HSPA12B,ITPR3,ITSN1,JUN,JUP,KHDRBS1,LARP1,LARP7,LIMD1,LPXN,LRRFIP1,MACF1,MAP4,MAP4K4,MAST4,MIB2,MTA1,MTMR12,MTOR,MYCT1,MYL9,NAA10,NAV1,NES,NIPBL,NUFIP2,OSBPL11,P4HA3,PA2G4,PABPN1,PAFAH1B2,PARP4,PCM1,PDLIM7,PFDN4,PHLDB2,PHRF1,PIKFYVE,PKN1,PLEC,PLEKHG5,PNN,PPFIBP1,PRKD3,PRRC2A,PUM1,PXN,RAB11FIP5,RABL6,RANBP3,RETREG2,RIN2,RIOK2,ROCK2,RPLP2,RPRD2,RRAGC,RRM2,SART1,SASH1,SCAF11,SCRIB,SEPT2,SHANK3,SIRT1,SMN1/SMN2,SMTN,SNTB2,SPECC1L,SYNPO,TBC1D9B,TJP2,T

			MSB10/TMSB4X,TMX1,TNIK,TNKS1BP1,TOP2A,TRIM28,TRIM56,TXLNA,UBE2O,USH2A,USP8,VIM,WASHC2A/WASHC2C,WDR44,XPO6
Cancer,organismal injury and abnormalities	Development of carcinoma	0.0005	ACVRL1,ADD3,AFAP1,AHNAK,ANXA1,ANXA2,AP3D1,APPL1,ARHGEF2,ARHGEF6,ATG16L1,ATG9A,ATP2B1,ATRX,ATXN2L,BIN1,BRD8,CAVIN1,CCAR2,CDK16,CEP170,CHCHD3,COPB2,CTNNA1,CTTNBP2NL,DDHD1,EEF2K,EIF5B,ERCC5,ERRFI1,FGD5,FKBP15,FLII,FLNB,G3BP2,GNG12,GORASP2,HDGF,HSPA12B,ITPR3,ITSN1,JUN,JUP,KHDRBS1,LARP1,LARP7,LIMD1,LPXN,LRRFIP1,MACF1,MAP4,MAP4K4,MAST4,MTA1,MTMR12,MTOR,MYCT1,MYL9,NAV1,NES,NIPBL,OSBPL3,PA2G4,PARP4,PCM1,PDLIM7,PHLDB2,PHRF1,PIKFYVE,PKN1,PLEC,PLEKHG5,PNN,PPFIBP1,PRRC2A,PUM1,PXN,RAB11FIP5,RAB12,RABL6,RANBP3,RIN2,RIOK2,ROCK2,RPLP2,RPRD2,RRM2,SART1,SASH1,SCAF11,SCRIB,SHANK3,SMTN,SPECC1L,SYNPO,TBC1D9B,TJP2,TNIK,TNKS1BP1,TOP2A,TRIM28,TXLNA,USH2A,USP8,VIM,WASHC2A/WASHC2C,WDR44,XPO6
Cancer,gastrointestinal disease,organismal injury and abnormalities	Gastrointestinal adenocarcinoma	0.0007	ACVRL1,ADD3,AFAP1,AHNAK,ANXA1,AP3D1,APPL1,ARHGEF2,ARHGEF6,ATG16L1,ATG9A,ATP2B1,ATRX,ATXN2L,BAG3,BRD8,CAAP1,CAVIN1,CCAR2,CCDC86,CDK16,CEP170,CHCHD3,COPB2,CTNNA1,CTTNBP2NL,DBNL,DDHD1,DDX55,EEF2K,EIF5B,ERCC5,ERRFI1,FAM114A1,FAM129B,FGD5,FKBP15,FLII,FLNB,FOXK1,G3BP2,GATAD2B,GNG12,GORASP2,HDAC7,HDGF,HSPA12B,ITPR3,ITSN1,JUN,JUP,KHDRBS1,LARP1,LARP7,LIMD1,LPXN,LRRFIP1,MACF1,MAP4,MAP4K4,MAST4,MIB2,MTA1,MTMR12,MTOR,MYCT1,MYL9,NAV1,NES,NIPBL,NUFIP2,OSBPL11,OSBPL3,PAFAH1B2,PARP4,PCM1,PDLIM7,PFDN4,PHLDB2,PHRF1,PIKFYVE,PKN1,PLEC,PLEKHG5,PNN,PPFIBP1,PRKD3,PRRC2A,PUM1,PXN,RAB11FIP5,RAB12,RABL6,RANBP3,RETREG2,RIN2,RIOK2,ROCK2,RPLP2,RPRD2,SASH1,SCAF11,SCRIB,SHANK3,SIRT1,SMTN,SNTB2,SPECC1L,SYNPO,TBC1D9B,TJP2,TMX1,TNIK,TNKS1BP1,TOP2A,TRIM28,TRIM56,TXLNA,UBE2O,USH2A,USP8,VIM,WDR44,XPO6

*Find names of proteins in Supplementary Table S7.

Supplementary Table S12. Top diseases and functions in networks of phosphoproteins differentially expressed in PAH PAEC

Networks	Top Diseases and Functions	Focus Molecules#	Molecules* in Network
1	RNA post-transcriptional modification, cellular development, cellular growth and proliferation	21	**AHNAK, ANXA2**, **ARHGEF2**, **CTNNA1**, **FLII**,G3BP1,**G3BP2**, **HDGF**, HNRNPR, ILK, **JUN**, **KHDRBS1**, **LRRFIP1**, **MAP4**, MAPK7, MSN, **PA2G4**, **PABPN1**, **PKN1**, **PLEC**, **PNN**, PPM1D, PPP2R1A, PTGES, **PXN**, RASA1, RPLP0, RPLP2, **SCRIB**, **SMN1/SMN2**,SRSF1,SYNCRIP, TRAF7, **VIM**, WDR5
2	Cancer, organismal injury and abnormalities, endocrine system disorders	15	**ACACA**, AKT1S1, **APPL1**, **CCAR2**, CDH4, CREBZF,CTNNB1,**EIF5B**, ENO1, GTF3C1, **JUP**, KDM5B, LANCL2, **LARP1**, **MTA1**, **MTOR**, **NIPBL**, PSME3, PTPRF, **PUM1**, **RABL6**, RALB, RAN, RB1CC1, RPTOR, **RRAGC**, RUVBL2, **SIRT1**, SMC1A, SREBF1, SREBF2, **TOP2A**, TOP2B, **TXLNA**, VPS39
3	Cancer, hematological disease, immunological disease	15	**ANXA2**, **ARHGEF2**, **ATP2B1**, **BRD8**, DUSP4, FBLIM1, **FLNB**, FZD8, **GORASP2**, HPGD, HSP90AA1, **LPXN**, LTBP1, MKI67, **NIFK**, **PCM1**, POLE2, PRIM1, PTX3, RALB, RGS2, **SEC22B**, SERPINH1, SMARCA4, **SMTN**, SOX4, STX16, STX6, **SYNPO**, TGFB1, **TMSB10/TMSB4X**, TNF, **TOP2A**, TP53, **VAMP4**
4	Cellular development, cellular growth and proliferation, gene expression	13	**AFAP1**, CBX3, CCND1, CDKN1C, DDX17, DDX5, **ERRFI1**, ETV5, F2, **FAM129B**, **FOXK1**, G3BP1, **GATAD2B**, GDF15, GPER1, KMT2A, KMT2E, LASP1, **MACF1**, MUC1, **MYCT1**, NRIP1, OGT, PFKP, PGR, PPRC1, RNF2, RUVBL2, **SNTB2**, **TJP2**, **TNIK**, **TRIM28**, TRPC1, **UBE2O**, **WDR44**
5	Hematological system development and function, lymphoid tissue structure and development, organ morphology	13	**ADD3**, **ANXA1**, **ATG16L1**, CAV2, **CAVIN1**, **CAVIN2**, CAVIN3, CFLAR, **DBNL**, DNAJC15, EPAS1, **ERCC5**, GADD45A, **HNRNPA0**, HSP90AA1, IKBKG, IL7, IL7R, IRF1, ITGB2, LEP, MAPK7, **MIB2**, **MYL9**, **NAV1**, NEUROG1, NR3C1, **PARP4**, PDLIM2, PDPK1, RBM5, **SCAF11**, SMAD4, TIMP3, TNFRSF10A
6	Cell death and survival, cellular development, cellular growth and proliferation	13	**ACVRL1**, **ATG9A**, BAX, BCL2L1, BMP2, DNM1L, **EEF2K**, HIF1A, HK2, IL22, **ITPR3**, **ITSN1**, KRAS, **LIMD1**, MST1R, **NES**, PDGFA, PDK1, **PDLIM7**, PHLPP1, PRKCD, PRKCE, PRKCZ, **PRKD3**, RACK1, RB1, **RIN2**, **RRM2**, **SART1**, SLC2A1, SP1, **TOP2A**, TRPC1, YWHAE, ZNF148
7	Cellular assembly and organization, cellular function and maintenance, cell morphology	12	**ARHGEF2**, **ARHGEF6**, ARHGEF7, **CEP170**, **COPB2**, CTTNBP2, **CTTNBP2NL**, ERG, ESR1, **FAM114A1**, **HDAC7**, MARK2, MARK3, MOB4, NFKB1, NR1D1, NUPR1, **PCNP**, **PFDN4**, **PHLDB2**, PLK3, PPP2CA, PPP2R1B, PRKCI, PTX3, **RAB11FIP5**, SAFB, SAFB2, **SHANK3**, SREBF2, STRIP1, STRN, STRN3, TP53BP2, YWHAG
8	Cell death and survival, organismal development, infectious disease	12	AGTR1, **ANXA2**, **AP3D**1, **ATRX**, CANX, **CHCHD3**, **CHMP3**, CLTC, EGFR, ERBB3, FASN, HBEGF, HNRNPA2B1, IGF2R, ILF3, **ITPRID2**, KCNH2, LGALS3, LONP1, **MAP4K4**, **MAST4**, **NAA10**, PDIA3, **PIKFYVE**, PKM, POSTN, SNX1, SNX2, SRSF1, STAMBP, **TMX1**, TRAP1, **WASHC2A/WASHC2C**, WASHC5, XRCC5
9	Cell death and survival, cellular function and maintenance, cell morphology	9	ACTB, APP, **ATXN2L**, BACE1, **BAG3**, BAG4, **BIN1**, BSG, DDX6, EGFR, HK2, HSF1, HSP90AA1, HSPA5, HSPA8, HSPA9, IL1B, IL7, MAPT, NR3C1, **NUFIP2**, **PAFAH1B2**, PRKCA, PSEN1, **ROCK2**, **SEPT2**, SNCA, SPHK1, STAU1, SUMO2, SYK, TP53, **USP8**, **XPO6**, XRN2

#Differentially expressed proteins; *Find names of proteins in bold in Supplementary Table S7.

Supplementary Table S13. 670 Mitochondrial proteins identified in PAH PAEC and healthy control PAEC

Protein	Gene	Fold Change	*P*-value
Alanine--tRNA ligase, mitochondrial	AARS2	0.9042	0.2980
Multidrug resistance-associated protein 1	ABCC1	0.8559	0.2753
ATP-binding cassette sub-family D member 3	ABCD3	0.8303	0.1360
ATP-binding cassette sub-family E member 1	ABCE1	1.0621	0.6061
Mycophenolic acid acyl-glucuronide esterase, mitochondrial	ABHD10	0.9151	0.4560
X-ray repair cross-complementing protein 6	XRCC6	1.1089	0.4639
Tyrosine--tRNA ligase, cytoplasmic	YARS	0.9587	0.7117
Tyrosine--tRNA ligase, mitochondrial	YARS2	1.3580	0.0310
14-3-3 protein epsilon	YWHAE	0.9877	0.8819
14-3-3 protein theta	YWHAQ	0.8930	0.3490

Fold Change, PAH to healthy controls; *P*-value, PAH *vs*. healthy controls.

Supplementary Table S14. 45 mitochondrial proteins differentially expressed in PAH PAEC compared with healthy control PAEC (all *P* < 0.05)

**Gene***	**Mitochondria Location**	**Up regulated (PAH/ Controls)**	***P*-value**	**Gene**	**Mitochondria Location**	**Down regulated (PAH/ Controls)**	***P*-value**
MTHFD1L	Mitochondrion	1.7884	0.0404	NOS3	Mitochondrion	0.2346	0.0302
MRPS31	Inner membrane, matrix, mitochondrial ribosome	1.7364	0.0017	EBP	Mitochondrion	0.3469	0.0018
MRPS7	Inner membrane, matrix, mitochondrial ribosome	1.5766	0.0259	MTCH1	Inner membrane	0.5459	0.0145
GOLPH3	Intermembrane space	1.5519	0.0496	MGST1	Inner membrane, outer membrane	0.5780	0.0160
NDUFB7	Inner membrane, intermembrane space	1.5018	0.0009	GSN	Mitochondrion	0.6440	0.0218
CASP4	Mitochondrion	1.4773	0.0363	MGARP	Outer membrane	0.6358	0.0410
TM9SF4	Mitochondrion	1.4288	0.0219	TCIRG1	Mitochondrion	0.6486	0.0057
NME1	Outer membrane	1.3751	0.0261	ACAT2	Mitochondrion	0.6976	0.0019
PPIF	Inner membrane, matrix	1.3687	0.0136	RMDN3	Outer membrane	0.6986	0.0484
ECI1	Inner membrane, matrix	1.3593	0.0045	SOD1	Inner membrane, matrix	0.7019	0.0432
YARS2	Matrix	1.3580	0.0310	AKT1	Mitochondrion	0.7279	0.0187
ARMCX3	Outer membrane	1.3352	0.0400	CTSL	Mitochondrion	0.7321	0.0100
PPID	Mitochondrion	1.3069	0.0242	DPYSL2	Mitochondrion	0.7269	0.0490
SHMT2	Inner membrane, intermembrane space, matrix	1.3038	0.0366	PMPCA	Inner membrane, matrix	0.7313	0.0320
RPL3	Mitochondrion	1.2822	0.0468	STAT1	Mitochondrion	0.7344	0.0095
CLPP	Matrix	1.2678	0.0134	IDI1	Mitochondrion	0.7392	0.0199
STOML2	Inner membrane, intermembrane space	1.2621	0.0276	MAPK1	Mitochondrion	0.7711	0.0109
HSPA5	Mitochondrion	1.2485	0.0488	SLC25A1	Inner membrane	0.7732	0.0381
MRPL23	Inner membrane, matrix, mitochondrial ribosome	1.2408	0.0490	CDIPT	Mitochondrion	0.7989	0.0379
MRPS28	Inner membrane, matrix, mitochondrial ribosome	1.2314	0.0385	LAMC1	Mitochondrion	0.8019	0.0230
HSP90B1	Mitochondrion	1.2167	0.0492	CAPN1	Mitochondrion	0.8206	0.0327
GRPEL1	Matrix	1.2123	0.0476	MOGS	Mitochondrion	0.8356	0.0339
ALDH18A1	Inner membrane	1.2121	0.0270				

*Find names of proteins in Supplementary Table S1; *P*-value, PAH *vs*. healthy controls.

Supplementary Table S15. 366 Mitochondrial phosphopeptides identified in PAH PAEC and healthy control PAEC

Protein	Gene	Peptide Sequence	#	Positions[&]	Fold Change	*P*-value
Multidrug resistance-associated protein 1	ABCC1	QISSSSSYSGDISR	800	1	0.8591	0.4231
Tyrosine-protein kinase ABL1	ABL1	GQGESDPIDHEPAVSPIIPR	569	1	0.9006	0.5587
Acetyl-CoA carboxylase 1;Biotin carboxylase	ACACA	FIIGSVSEDNSEDEISNIVK	23	2	0.4764	0.0014
Acetyl-CoA carboxylase 1;Biotin carboxylase	ACACA	FIIGSVSEDNSEDEISNIVK	25	1;2	1.0839	0.8038
Acetyl-CoA carboxylase 1;Biotin carboxylase	ACACA	FIIGSVSEDNSEDEISNIVK	29	1;2	1.2002	0.5432
Vimentin	VIM	ETNIDSIPIVDTHSKR	438	1	1.1317	0.6103
Vimentin	VIM	DGQVINETSQHHDDIE	458	1	0.3723	0.0617
Vimentin	VIM	DGQVINETSQHHDDIE	459	1	1.1244	0.6275
Yorkie homolog	YAP1	GDSETDIEAIFNAVMNPK	61	1	0.8452	0.5781
Yorkie homolog	YAP1	QASTDAGTAGAITPQHVR	109	1	1.0318	0.8920

#, number of phosphorylation sites on that specific peptide; [&]The amino acid residue within the protein that is phosphorylated; Fold Change, PAH to healthy controls; *P*-value, PAH *vs*. healthy controls.

Supplementary Table S16. 18 mitochondrial phosphopeptides differentially expressed between PAH PAEC and healthy control PAEC (all *P* < 0.05)

Gene*	Positions[&]	Mitochondria Location	Up regulated (PAH/ Controls)	*P*-value	Proteome (Fold Change)	Gene	Positions[&]	Mitochondria Location	Down regulated (PAH/ Controls)	*P*-value	Proteome (Fold Change)
CHCHD3	50	Inner membrane	2.7993	0.0072	0.9942	MTOR	1166	Mitochondrial membrane	0.1981	0.0019	
PLEC	4396	Mitochondrion	2.1967	0.0313	1.1045	ITPR3	1832	Mitochondrion	0.4299	0.0100	0.8943
VIM	63	Mitochondrion	2.1504	0.0147	0.9247	RPLP2	79	Mitochondrion	0.4312	0.0247	0.9449
TBC1D9B	435	Mitochondrion	2.1215	0.0357	0.9332	ANXA1	5	Mitochondrial membrane	0.4542	0.0057	0.9902
JUN	62	Mitochondrion	2.0110	0.0254		ACACA	23	Mitochondrion	0.4764	0.0014	0.9836
RAB11FIP5	359	Outer membrane	1.8613	0.0262		MYH10	1952	Mitochondrion	0.5205	0.0047	0.8854
DNAJC5	10	Mitochondrion	1.5948	0.0232		PARP4	1504	Mitochondrion	0.5734	0.0453	0.8526
						ATG16L1	143	Mitochondrion	0.6141	0.0047	
						SIRT1	16	Mitochondrion	0.6633	0.0453	
						TOP2A	1247	Mitochondrion	0.6725	0.0182	
						TP53BP1	830	Mitochondrion	0.7059	0.0207	1.1083

*Find names of proteins and peptide sequence in Supplementary Table S7; [&]The amino acid residue within the protein that is phosphorylated; *P*-value, PAH *vs*. healthy controls; Proteome (fold change), ratio of PAH to healthy controls.

Supplementary Table S17. Biological functions of mitochondrial proteins differentially expressed in PAH PAEC

Pathways	*P*-value of Overlap	Molecules*
Organonitrogen compound biosynthetic process	<0.0001	AKT1,ALDH18A1,GOLPH3,MGST1,MOGS,MRPS28,MRPS31,MRPS7,MTHFD1L,NME1,RPL23L,RPL3,SHMT2,SLC25A1,STOML2,TCIRG1,YARS2
Organonitrogen compound metabolic process	<0.0001	AKT1,ALDH18A1,CAPN1,CASP4,CDIPT,CLPP,CTSL,GOLPH3,GSN,HSP90B1,HSPA5,LAMC1,MAPK1,MGST1,MOGS,MRPS28,MRPS31,MRPS7,MTHFD1L,NDUFB7,NME1,NOS3,PMPCA,PPID,PPIF,RPL23L,RPL3,SHMT2,SLC25A1,SOD1,STOML2,TCIRG1,YARS2
Organic substance metabolic process	<0.0001	ACAT2,AKT1,ALDH18A1,CAPN1,CASP4,CDIPT,CLPP,CTSL,DPYSL2,EBP,ECI1,GOLPH3,GSN,HSP90B1,HSPA5,IDI1,LAMC1,MAPK1,MGST1,MOGS,MRPS28,MRPS31,MRPS7,MTHFD1L,NDUFB7,NME1,NOS3,PMPCA,PPID,PPIF,RPL23L,RPL3,SHMT2,SLC25A1,SOD1,STAT1,STOML2,TCIRG1,YARS2
Small molecule metabolic process	0.0001	ACAT2,AKT1,ALDH18A1,CDIPT,EBP,ECI1,IDI1,MAPK1,MTHFD1L,NDUFB7,NME1,NOS3,SHMT2,SLC25A1,STOML2,TCIRG1,YARS2
Cellular metabolic process	0.0001	ACAT2,AKT1,ALDH18A1,CAPN1,CDIPT,CLPP,CTSL,DPYSL2,ECI1,GOLPH3,GSN,HSP90B1,HSPA5,IDI1,LAMC1,MAPK1,MGST1,MOGS,MRPS28,MRPS31,MRPS7,MTHFD1L,NDUFB7,NME1,NOS3,PMPCA,PPID,PPIF,RPL23L,RPL3,SHMT2,SLC25A1,SOD1,STAT1,STOML2,TCIRG1,YARS2
Primary metabolic process	0.0001	ACAT2,AKT1,ALDH18A1,CAPN1,CASP4,CDIPT,CLPP,CTSL,DPYSL2,EBP,ECI1,GOLPH3,GSN,HSP90B1,HSPA5,IDI1,LAMC1,MAPK1,MOGS,MRPS28,MRPS31,MRPS7,MTHFD1L,NDUFB7,NME1,NOS3,PMPCA,PPID,PPIF,RPL23L,RPL3,SHMT2,SLC25A1,STAT1,STOML2,TCIRG1,YARS2
Cellular component organization	0.0001	AKT1,CLPP,CTSL,DPYSL2,GOLPH3,GRPEL1,GSN,HSP90B1,LAMC1,MAPK1,MGARP,MGST1,MRPS28,MRPS31,MRPS7,MTCH1,NDUFB7,NOS3,PMPCA,PPID,PPIF,RPL23L,RPL3,SHMT2,SOD1,STOML2,TCIRG1,TM9SF4
Nitrogen compound metabolic process	0.0003	AKT1,ALDH18A1,CAPN1,CASP4,CDIPT,CLPP,CTSL,DPYSL2,GOLPH3,GSN,HSP90B1,HSPA5,LAMC1,MAPK1,MGST1,MOGS,MRPS28,MRPS31,MRPS7,MTHFD1L,NDUFB7,NME1,NOS3,PMPCA,PPID,PPIF,RPL23L,RPL3,SHMT2,SLC25A1,SOD1,STAT1,STOML2,TCIRG1,YARS2
Protein metabolic process	0.0003	AKT1,CAPN1,CASP4,CLPP,CTSL,GOLPH3,GSN,HSP90B1,HSPA5,LAMC1,MAPK1,MOGS,MRPS28,MRPS31,MRPS7,PMPCA,PPID,PPIF,RPL23L,RPL3,SHMT2,STOML2,TCIRG1,YARS2
Biosynthetic process	0.0005	ACAT2,AKT1,ALDH18A1,CDIPT,EBP,GOLPH3,IDI1,MAPK1,MGST1,MOGS,MRPS28,MRPS31,MRPS7,MTHFD1L,NME1,NOS3,RPL23L,RPL3,SHMT2,SLC25A1,SOD1,STAT1,STOML2,TCIRG1,YARS2
Protein-containing complex subunit organization	0.0005	CLPP,GSN,LAMC1,MGST1,MRPS28,MRPS31,MRPS7,NDUFB7,PPID,RPL23L,RPL3,SHMT2,STOML2,TCIRG1,TM9SF4
Cellular protein metabolic process	0.0009	AKT1,CLPP,CTSL,GSN,HSP90B1,HSPA5,LAMC1,MAPK1,MOGS,MRPS28,MRPS31,MRPS7,PMPCA,PPID,PPIF,RPL23L,RPL3,SHMT2,STOML2,TCIRG1,YARS2
Cellular process	0.0014	ACAT2,AKT1,ALDH18A1,CAPN1,CASP4,CDIPT,CLPP,CTSL,DPYSL2,EBP,ECI1,GOLPH3,GRPEL1,GSN,HSP90B1,HSPA5,IDI1,LAMC1,MAPK1,MGARP,MGST1,MOGS,MRPS28,MRPS31,MRPS7,MTCH1,MTHFD1L,NDUFB7,NME1,NOS3,PMPCA,PPID,PPIF,RMDN3,RPL23L,RPL3,SHMT2,SLC25A1,SOD1,STAT1,STOML2,TCIRG1,TM9SF4,YARS2
Transport	0.0014	AKT1,CAPN1,DPYSL2,EBP,GOLPH3,GRPEL1,GSN,HSP90B1,HSPA5,MAPK1,MGARP,MGST1,NME1,PMPCA,PPID,PPIF,RPL3,SLC25A1,SOD1,STOML2,TCIRG1,TM9SF4
Localization	0.0016	AKT1,ARMCX3,CAPN1,DPYSL2,EBP,GOLPH3,GRPEL1,GSN,HSP90B1,HSPA5,LAMC1,MAPK1,MGARP,MGST1,NME1,NOS3,PMPCA,PPID,PPIF,RPL3,SLC25A1,SOD1,STOML2,TCIRG1,TM9SF4

Cellular biosynthetic process	0.0018	AKT1,ALDH18A1,CDIPT,GOLPH3,IDI1,MAPK1,MGST1,MOGS,MRPS28,MRPS31,MRPS7,MTHFD1L,NME1,NOS3,RPL23L,RPL3,SHMT2,SLC25A1,SOD1,STAT1,STOML2,TCIRG1,YARS2
Organic substance biosynthetic process	0.0022	ACAT2,AKT1,ALDH18A1,CDIPT,EBP,GOLPH3,IDI1,MAPK1,MGST1,MOGS,MRPS28,MRPS31,MRPS7,MTHFD1L,NME1,RPL23L,RPL3,SHMT2,SLC25A1,STAT1,STOML2,TCIRG1,YARS2
Immune system process	0.0043	AKT1,CAPN1,CASP4,CTSL,EBP,GOLPH3,GSN,HSP90B1,MAPK1,MGST1,SHMT2,SOD1,STAT1,STOML2,TCIRG1
Cellular response to chemical stimulus	0.0044	AKT1,CASP4,CTSL,GSN,HSP90B1,HSPA5,MAPK1,MGARP,MGST1,NME1,NOS3,PPIF,RPL3,SOD1,STAT1,TCIRG1
Response to organic substance	0.0070	AKT1,CASP4,CTSL,GSN,HSP90B1,HSPA5,MAPK1,MGARP,MGST1,NME1,NOS3,RPL3,SHMT2,SOD1,STAT1,TCIRG1
Response to chemical	0.0078	AKT1,CASP4,CTSL,DPYSL2,EBP,GSN,HSP90B1,HSPA5,MAPK1,MGARP,MGST1,NME1,NOS3,PPIF,RPL3,SHMT2,SLC25A1,SOD1,STAT1,TCIRG1

*Find names of proteins in Supplementary Table S1.

Supplementary Table S18. Top biological processes of mitochondrial phosphoproteins differentially expressed in PAH PAEC

Pathways	*P*-value of Overlap	Molecules*
Response to stress	0.0172	ANXA1,ATG16L1,ITPR3,JUN,MTOR,MYH10,PARP4,SIRT1,TOP2A, TP53BP1,VIM
Cellular response to stress	0.0172	ANXA1,JUN,MTOR,PARP4,SIRT1,TOP2A,TP53BP1
Cellular component organization	0.0172	ACACA,ANXA1,ATG16L1,CHCHD3,ITPR3,JUN,MTOR,MYH10,PLEC,SIRT1,TOP2A,TP53BP1,VIM
Negative regulation of biological process	0.0172	ANXA1,CHCHD3,DNAJC5,ITPR3,JUN,MTOR,RAB11FIP5,RPLP2,SIRT1,TOP2A,TP53BP1,VIM
Regulation of biological quality	0.0172	ACACA,ANXA1,DNAJC5,ITPR3,JUN,MTOR,MYH10,RAB11FIP5,SIRT1,VIM
Cellular localization	0.0172	ACACA,ATG16L1,DNAJC5,ITPR3,MYH10,RAB11FIP5,RPLP2,TBC1D9B
Establishment of localization in cell	0.0172	ACACA,DNAJC5,ITPR3,MYH10,RAB11FIP5,RPLP2,TBC1D9B
Negative regulation of cellular process	0.0192	ANXA1,CHCHD3,DNAJC5,ITPR3,JUN,MTOR,RAB11FIP5,SIRT1,TOP2A,TP53BP1,VIM
Regulation of cellular component organization	0.0192	ANXA1,JUN,MTOR,MYH10,SIRT1,TBC1D9B,TOP2A,VIM
Regulation of transport	0.0192	ANXA1,ITPR3,MTOR,MYH10,RAB11FIP5,SIRT1,TBC1D9B

*Find names of proteins in Supplementary Table S7.

Supplementary Table S19. Clinical features of study participants for metabolomics

	**Controls (*n* = 12)**	**PAH (*n* = 30)**	***P*-value**
Mean age, yr	42 ± 3	44 ± 2	0.5
Gender, M/F	2/10	9/21	0.3
Ethnicity, W/AA/other	11/1/0	23/6/1	0.4
Cardiac output, l/min	3.8 ± 0.2	4.7 ± 0.2	0.01
PVR, wood unit	1.4 ± 0.1	2.7 ± 0.2	<0.0001
RVSP, mm Hg	25 ± 1	67 ± 4	<0.0001
O2 saturation, % of Hgb	98.6 ± 0.5	96.3 ± 0.4	0.002
6 minute walk distance, feet	NA	1513 ± 69	
Medications			
Phosphodiesterase type 5 inhibitor or stimulator of soluble guanylate cyclase, *n* (%)	NA	24 (80%)	
Endothelin receptor antagonist, *n* (%)	NA	19 (63%)	
Prostacyclin or prostacyclin receptor agonist, *n* (%)	NA	16 (53%)	

Mean ±SEM; *P*-value, PAH *vs*. controls; M, male; F, female; W, white; AA, African American; PVR, pulmonary vascular resistance; RVSP, right ventricular systolic pressure; Hgb, hemoglobin; NA, not applicable; Phosphodiesterase type 5 inhibitors and stimulators of soluble guanylate cyclase include tadalafil and sildenafil; endothelin receptor antagonist includes bosentan, ambrisentan and macitentan; prostacyclin and prostacyclin receptor agonist include treprostinil and epoprostenol [1,2,3].

Supplementary Table S20. Metabolite analysis in PAH versus healthy controls (PAHTCH Study)

Biochemical Name	Platform	Comp ID	Super Pathway	Fold Change	*P*-value
1-arachidonylglycerol (20:4)	LC/MS neg	34397	Lipid	1.1204	0.6445
1-dihomo-linolenylglycerol (20:3)	LC/MS neg	48341	Lipid	1.0472	0.6873
1-linoleoylglycerol (18:2)	LC/MS neg	27447	Lipid	1.0116	0.8344
1-methylhistidine	LC/MS neg	30460	Amino Acid	1.2692	0.1594
1-methylimidazoleacetate	LC/MS pos early	32350	Amino Acid	1.6121	0.0001
xanthine	LC/MS polar	3147	Nucleotide	1.4579	0.0758
xanthosine	LC/MS neg	15136	Nucleotide	1.6882	0.0488
xanthurenate	LC/MS neg	15679	Amino Acid	0.5325	0.1109
ximenoylcarnitine (C26:1)	LC/MS pos late	57517	Lipid	1.1944	0.2022
xylose	LC/MS polar	15581	Carbohydrate	0.6874	0.5558

Fold Change, PAH to healthy controls; *P*-value, PAH *vs*. healthy controls; NA, not available.

Supplementary Table S21. Purity of PAEC used for all experiments as assessed with CD31

PAEC	%CD31 Positive
Control 1*	99.2
Control 2*	99.9
Control 3	99.8
Control 4	97.2
Control 5	98.4
Control 6	99.7
Control 7	99.7
Control 8	99.7
Control 9	99.9
Control 10	99.9
Control 11	99.9
Control 12	96.8
Control 13*	99.2
Control 14*	97.1
PAH 1	97.1
PAH 2	97.5
PAH 3	97.8
PAH 4	97.4
PAH 5	97.4
PAH 6	98.7
PAH 7	98.0
PAH 8	99.1
PAH 9	97.1
PAH 10	98.8
PAH 11	99.9
PAH 12	98.7
PAH 13	97.1
PAH 14	97.5
PAH 15	99.0

*Commercially purchased

Supplementary Figure 1

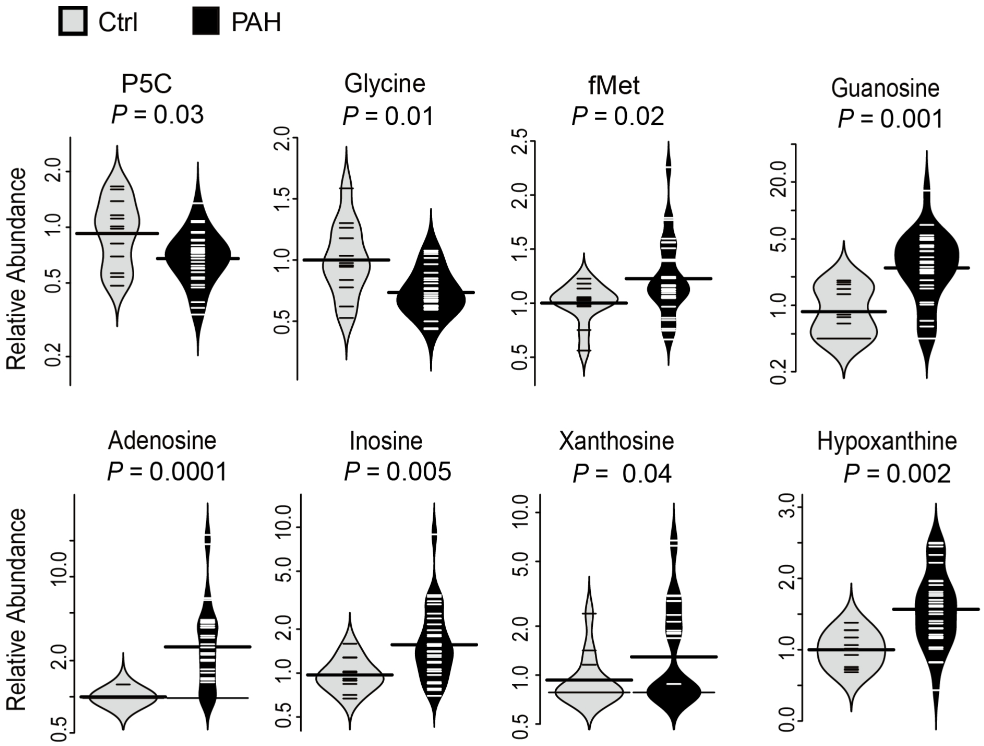

Supplementary Fig. S1 Nontargeted metabolites in PAH. Relative abundances of Δ1-pyrroline-5-carboxylate (P5C), glycine, N-formylmethionine (fMet), guanosine, adenosine, inosine, xanthosine, and hypoxanthine in plasma were significantly different between PAH (n = 30) and healthy controls (n =12). Beanplots were prepared using R 3.5.1.

Supplementary Figure 2

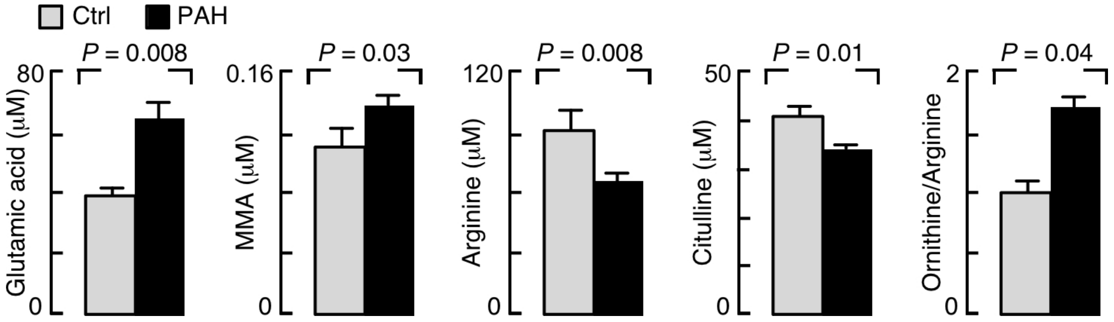

Supplementary Fig. S2 Targeted HPLC measures of metabolites in PAH. The concentration of glutamic acid, monomethylarginine (MMA), arginine, citrulline, and ratio of ornithine/arginine, a surrogate for arginase activity, in plasma were significantly different between PAH (*n* = 30) and healthy controls (*n* =12) (means ± SEM).

Supplementary Figure 3

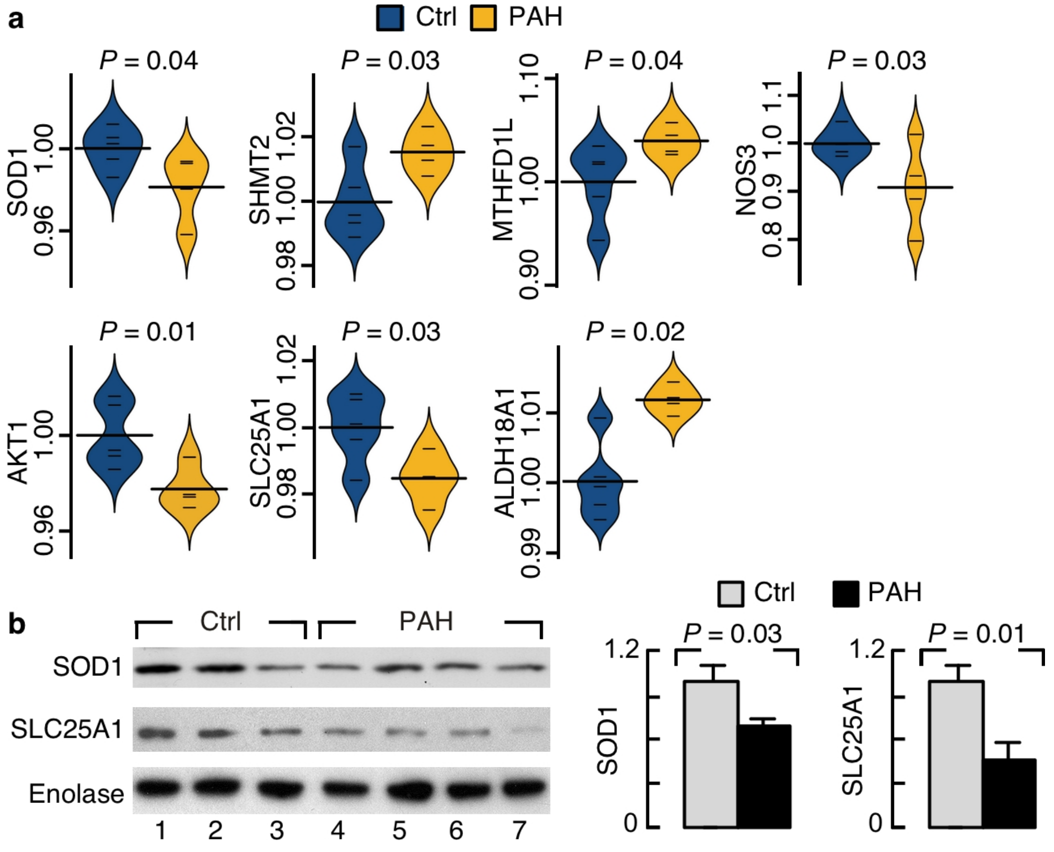

Supplementary Fig. S3 Mitochondrial protein expression in PAH. (**a**) Beanplots showing differential expression of the mitochondrial proteins SOD1, SHMT2, MTHFD1L, NOS3, AKT1, SLC25A1 and ALDH18A1 between PAH PAEC (n = 4) and healthy control PAEC (n = 5). (**b**) Decreased SOD1 and SLC25A1 protein expression was confirmed in PAH PAEC by Western blot. Enolase as a loading control. Replicate samples run on parallel gels are presented. Relative units (means ± SEM) in PAH PAEC and healthy control PAEC (SOD1, Ctrl n = 7, PAH n = 7; SLC25A1, Ctrl n = 5, PAH n = 4; two-tailed t-test). AKT1, RAC-alpha serine/threonine-protein kinase; ALDH18A1, delta-1-pyrroline-5-carboxylate synthase; MTHFD1L, monofunctional C1-tetrahydrofolate (THF) synthase; NOS3, endothelial nitric oxide synthase; SHMT2, serine hydroxymethyltransferase; SLC25A1, tricarboxylate transport protein; SOD1, superoxide dismutase [Cu-Zn].

Supplementary Figure 4

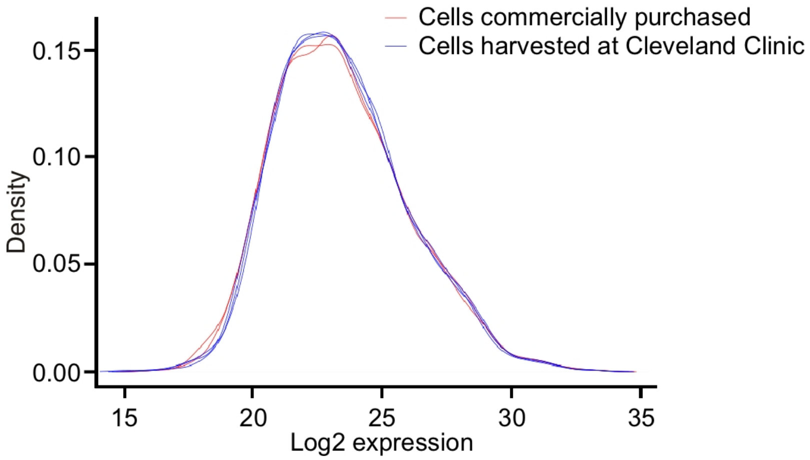

Supplementary Fig. S4 Overall expression of proteins was identical among control PAEC from commercially purchased (n = 2) and harvested at Cleveland Clinic (n = 3).

Supplementary Methods

Human samples. Plasma samples were obtained from 30 PAH patients and 12 healthy controls as part of the PAH Treatment with Carvedilol for Heart Failure (PAHTCH) trial (Cleveland Clinic IRB study number 11-1198) [1,2,3].

Flow cytometry. CellROX Green fluorogenic probe (Life Technologies) was used to quantify reactive oxygen species (ROS) by flow cytometry. Cell suspensions of PAEC in culture medium ($0.25 \times 10^6/100\ \mu L$) were stained with 5 μm probe per manufacturer's instructions. ROS generation was induced by incubation of cells with 100 μM menadione prior to CellROX staining. Cells treated with vehicle alone were used as a control to determine autofluorescence and to set gating boundaries for CellROX. Samples were acquired using an LSRII (Becton Dickinson) flow cytometer. CellROX Green was excited using a 488 nm laser line and fluorescence signals were collected using a 525/50 bandpass filter. 100,000 events were acquired.

Western blot analyses. Whole cell lysates were prepared as previously described [4]. Equal amounts of protein (80 μg) were loaded per lane. Protein was separated by electrophoresis on a 4–15% Tris-HCl (Bio-Rad Lab, Hercules, CA) and transferred onto polyvinylidene difluoride membranes (PVDF, Millipore Corporation, Bedford, MA). Amersham ECL Western blot detection reagents, Amersham Hyperfilm ECL (GE Healthcare Life Sciences) and Kodak M35 X-OMAT Automatic Processors (Rochester, NY) were used for detection of signals. Antibodies included rabbit anti-Enolase (sc-15343), SOD1 (sc-11407, Santa Cruz Biotechnology, Santa Cruz, CA) and SLC25A1 (Thermo Fisher Scientific, Waltham, MA, #PA5-42451) polyclonal Ab and anti-rabbit (NA9340)(GE Healthcare Life Sciences, Marlborough, MA) secondary Ab.

Amino acid analysis. Plasma amino acid concentrations were measured using HPLC (Agilent 1100 series HPLC; Agilent Technologies, Wilmington, DE), following *ortho*-phthalaldehyde derivatization using a fluorescent detector as described previously [5].

References

1. Farha, S., *et al*. Pulmonary arterial hypertension treatment with carvedilol for heart failure: a randomized controlled trial. *JCI Insight* **2**, (2017).
2. Cheong, H. I., *et al*. Endothelial Phenotype Evoked by Low Dose Carvedilol in Pulmonary Hypertension. *Front Cardiovasc Med* **5**, 180 (2018).
3. Saygin, D., *et al*. Metabolic and Functional Evaluation of the Heart and Lungs in Pulmonary Hypertension by Gated 2-[18F]-Fluoro-2-deoxy-D-glucose Positron Emission Tomography. *Pulm Circ* **7**, 428-438 (2017).
4. Xu, W., *et al*. STAT-1 and c-Fos interaction in nitric oxide synthase-2 gene activation. *Am J Physiol Lung Cell Mol Physiol* **285**, L137-148 (2003).
5. Comhair, S. A., *et al*. Metabolomic Endotype of Asthma. *J Immunol* **195**, 643-650 (2015).
